# A Compartmental Model Analysis of Integrative and Self-Regulatory Ion Dynamics in Pollen Tube Growth

**DOI:** 10.1371/journal.pone.0013157

**Published:** 2010-10-06

**Authors:** Junli Liu, Bernard M. A. G. Piette, Michael J. Deeks, Vernonica E. Franklin-Tong, Patrick J. Hussey

**Affiliations:** 1 School of Biological and Biomedical Sciences, University of Durham, Durham, United Kingdom; 2 Department of Mathematical Sciences, University of Durham, Durham, United Kingdom; 3 Biophysical Sciences Institute, University of Durham, Durham, United Kingdom; 4 School of Biosciences, University of Birmingham, Birmingham, United Kingdom; John Innes Centre, United Kingdom

## Abstract

Sexual reproduction in higher plants relies upon the polarised growth of pollen tubes. The growth-site at the pollen tube tip responds to signalling processes to successfully steer the tube to an ovule. Essential features of pollen tube growth are polarisation of ion fluxes, intracellular ion gradients, and oscillating dynamics. However, little is known about how these features are generated and how they are causally related. We propose that ion dynamics in biological systems should be studied in an integrative and self-regulatory way. Here we have developed a two-compartment model by integrating major ion transporters at both the tip and shank of pollen tubes. We demonstrate that the physiological features of polarised growth in the pollen tube can be explained by the localised distribution of transporters at the tip and shank. Model analysis reveals that the tip and shank compartments integrate into a self-regulatory dynamic system, however the oscillatory dynamics at the tip do not play an important role in maintaining ion gradients. Furthermore, an electric current travelling along the pollen tube contributes to the regulation of ion dynamics. Two candidate mechanisms for growth-induced oscillations are proposed: the transition of tip membrane into shank membrane, and growth-induced changes in kinetic parameters of ion transporters. The methodology and principles developed here are applicable to the study of ion dynamics and their interactions with other functional modules in any plant cellular system.

## Introduction

Higher plants reproduce sexually by using the male gametophyte (the pollen grain) to grow rapidly through sporophytic pistil tissue to effect double-fertilization of the embryo sac, and produce seed. This involves growth of a pollen tube using directional, polar tip growth. This involves a highly coordinated movement of vesicles bearing large amounts of new cell wall and plasma membrane materials to be integrated into the growing apical region. Tip growth is a specialized type of growth used by several cell types, including fungal hyphae and animal neuronal cells. In Angiosperms (the flowering plants) the pollen tube can traverse up to 30 centimetres of pistil tissue before encountering an ovule.

Molecular signals affect the position of the polarised growth site and consequently guide the direction of tip growth. Interpretation of these signals requires a plethora of proteins and other molecules (recently named the LENS: Localisation Enhancing Network, Self-sustaining [1]). An exchange of ions across the plasma membrane maintains stable cytosolic ion gradients relative to the growing tube tip. Ion flux, growth and cytoskeletal rearrangement occur at the tip in an oscillatory manner [2]–[4]. This oscillatory flux at the tip contrasts with the steady ion flux across the plasma membrane of the pollen tube shank [5]. Experimental manipulation of tip-associated calcium ion gradients can result in the re-polarisation of the LENS [6] suggesting an intimate relationship between ion flux and pollen tube growth and guidance. Protein ion transporters and ion channels regulate oscillatory and non-oscillatory flow at both the tip and shank; their influence on flux is likely to be dictated by their sub-cellular distribution, activation and gating properties.

The dynamics of ion transporters in cells have been the subject of both experimental and theoretical studies [7], [8]. In particular, a model which includes the activity of five transporters has been successfully used to study the oscillatory dynamics of ion transporters in plant cells [8], [9]. It has also been shown that model predictions can be validated by experimental measurements from various plant cells [10]. The wealth of information of the growth of pollen tubes makes this a tractable system for developing a model that will aid our understanding of polarized tip growth in general. Pollen tube growth can be experimentally separated into a “multi-compartment” system which allows the development of a compartmental model. The tip and shank in a pollen tube can be considered as two different compartments with distinct features, including a) differentially localised transporters, b) qualitatively different ion dynamics: oscillatory ion dynamics at the tip and non-oscillatory ion dynamics at the shank, c) polarised ion fluxes, d) stable ion gradients between the tip and shank and (e) an electrical current entering the tip and leaving at the shank [11], [12]. Although it is widely recognised that these features play important roles, little is known about how they are generated and how they are causally related. Specifically: How are the stable cytosolic ion gradients established? How does a pollen tube implement a strategy for responding to intracellular and extracellular perturbations? Are the oscillatory dynamics important for forming intracellular ion gradients? What are the roles of a current travelling along a pollen tube? What are the possible mechanisms for growth-induced oscillatory ion dynamics? While progress has been made in elucidating the interplay between ions specifically by accumulating data on the relevant transporters experimentally in *Arabidopsis* [12], [13], understanding the more intricate features of pollen tube development requires the development of a model at a systemic level [12].

In the context of ion flux, the growing pollen tube can approximately be divided into three regions with distinct properties [12], [14]: 1) the tip where net (electrical) current enters the cell with oscillatory dynamics, 2) the shank with net outward current at steady state, and 3) the large body. Here we show that pollen tube growth can be modelled by developing a three-compartment model (Data S1 in the Supporting Information). Based on the three-compartment model, we further develop a two-compartment model which mimicks the tip and shank compartments in a pollen tube. We demonstrate that this model not only reproduces experimental observations, but also develops novel insights into how a pollen tube evolves integrative and self-regulatory ion dynamics and the nature of the growth-induced mechanisms for oscillatory dynamics.

## Results

### Compartmental model for tip-shank interaction in the pollen tube

**Figure 1:**
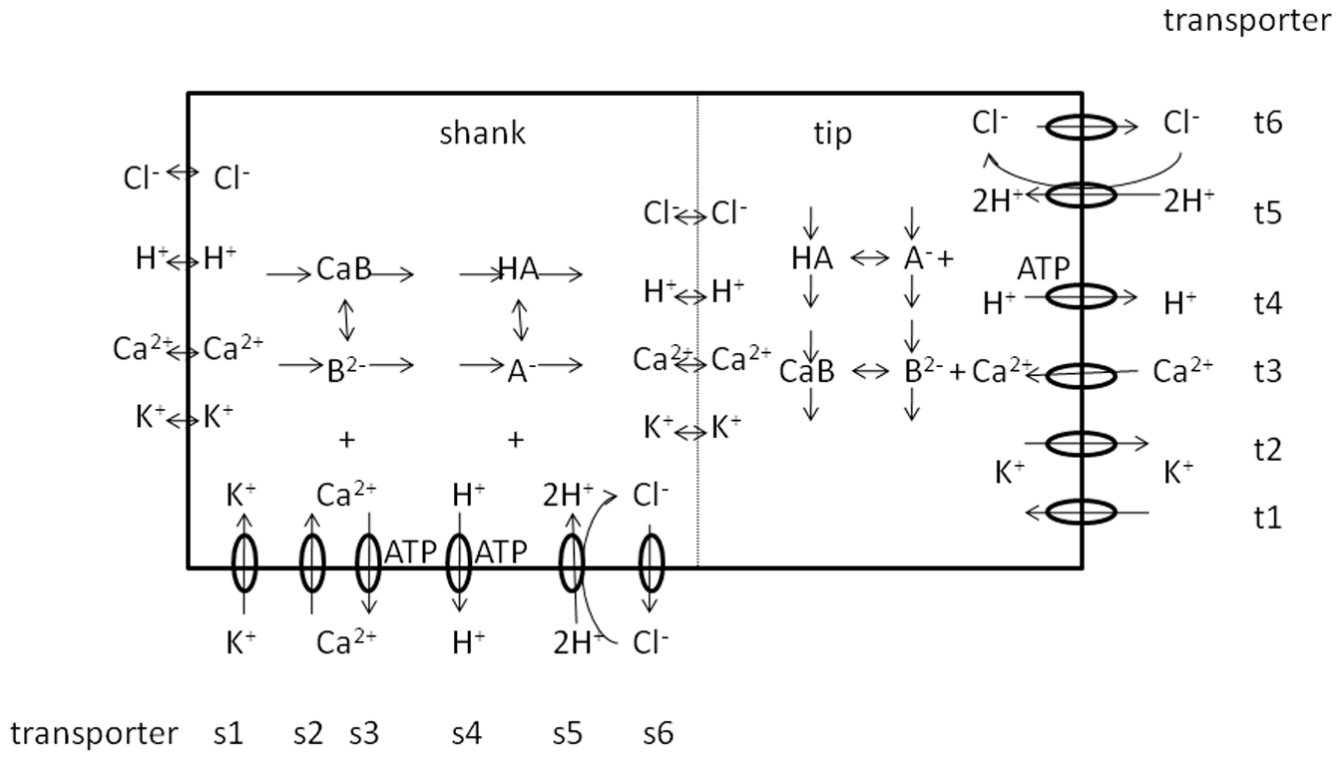
The model. A two-compartment model for investigating tip-shank interactions in pollen tube growth.

Following the principles for developing compartmental models which are described in the Data S1 in the Supporting Information and using the wealth of biological data on pollen tube growth, we have developed a tip-shank two-compartment model to study ion dynamics and growth and how these are related (Figure 1). The properties of the appropriate ion transporters that we have used in this study are described in Materials and Methods. The model construction process is as follows.

Experimentally, it is known that the tip and shank of a pollen tube possess the following main properties [11], [12]:

- ion fluxes across the plasma membrane at the tip and shank are polarised;
- intracellular ion gradients are established between the tip and shank;
- the pollen tube tip possesses the property of oscillatory ion and growth dynamics; and
- the pollen tube shank possesses the property of non-oscillatory ion and growth dynamics.

As described below, we initially define the volume of the shank compartment to be equal to the volume of the tip compartment. In this context the shank compartment corresponds to the “subapical region” in a growing pollen tube [5], [11]. Then we examine the effects of the tip and shank volume on ion dynamics. In addition, the model developed in this work is able to study the ion dynamics of a pollen tube with different sizes of tip and shank volumes as it explicitly includes volumes as variables. Therefore, the model can be generally applied to study the growth of a pollen tube in different plant species, as we will show below.

We develop the model based on the following major experimental observations. Firstly, calcium enters a pollen tube at the tip and leaves the pollen tube at the shank [11], [12]. Secondly, the abundance of proton ATPase transporters at the tip is much lower than that at the shank and reduces towards the apex of the tip [15]. Thirdly, although the potassium fluxes at the tip and shank remain debatable [11], [12], the consensus is that potassium is transported via channels at both the tip and the shank [11], [12]. Furthermore, it is known that chloride is also transported via channels at both the tip and shank [11], [12], [16]. Based on these biological investigations and resulting data, we have developed a tip-shank two-compartment model. The model assumes that both tip and shank are homogenous compartments and that they exchange ions by cyclosis and/or by diffusion. At either the tip or the shank, there are six transporters (Figure 1). At both tip and shank, an electrogenic H^+^ ATPase pump is included, but different parameters for this pump are used at the tip and shank for the following reasons: a) the H^+^ ATPase pump is considered to be a ubiquitous plasma membrane pump [17], [18] and previous models have shown its importance in plant cells [8]–[10]; b) experimental evidence indicates that the abundance of H^+^ ATPase at the tip is much lower than that at the shank and reduces towards the apex of the tip [15]. At both the tip and shank, a Cl^−^-2H^+^symporter is included. This is because such a symporter mediates anion uptake into plant cells [19], [20] and it has been shown that Cl^−^ are transported using channels at both the tip and the shank of a pollen tube [11], [12], [16], [21], [22]. At the tip, both K^+^ inward and outward rectifying channels are included, as there are experimental data showing that K^+^ are transported in and out of a pollen tube at the tip [11], [12], [23].

In the process of developing the model, we demonstrate that both K^+^ inward and outward rectifying channels can also be included at the shank even though only K^+^ inward channels have been reported experimentally. By removing the K^+^ outward rectifying channel at the shank our model shows that there would be no marked effect on ion concentrations and fluxes. As a result we have only included a K^+^ inward rectifying channel at the shank. At both the tip and shank, a calcium channel is included. Experimentally, it has been shown that calcium channels exist at the tip [11], [12]. However in the development of our model it was necessary to include a calcium channel at the shank in order for the system to reach experimentally observed ion gradients. At the shank, a calcium ATPase pump is included consistent with experimental observation [11], [12]. A calcium ATPase pump can also been included at the tip, however, removal of calcium ATPase pump does not affect our conclusion. Therefore, we have not included calcium ATPase pump in our model.

### Ion dynamics at both the tip and shank

Based on the two-compartment model (figure 1, and Data S1 in the Supporting Information), the ion dynamics at both tip and shank are described as follows.

At the tip,
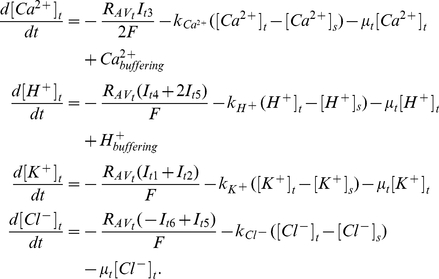
(1)At the shank,
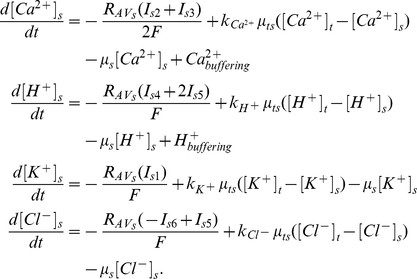
(2)In the right hand of equations (1) and (2), the first term describes the transport of ions through the membrane; the second term describes the exchange of ions between the tip and shank; the third term describes the effects of volume changes on the ion concentrations. Initially we assume that the volume of both the tip and shank compartments are constant, therefore, the third term is zero. For Ca^2+^ and H^+^, the fourth term (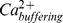 or 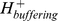) describes the buffering of Ca^2+^ and H^+^ respectively. 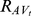 and 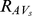 are the surface to volume ratio for describing the density of transporters at the membrane surface of the tip and shank compartments, and they are set to be equal throughout this work. 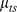 is the volume ratio between tip and shank, and it is set to be 1 except where we examine the effects of growth on changes in volume. The calculations of the current density of all ion transporters and their voltage-gating properties are summarised in Materials and Methods.

### Ion gradients and oscillating dynamics

**Figure 2:**
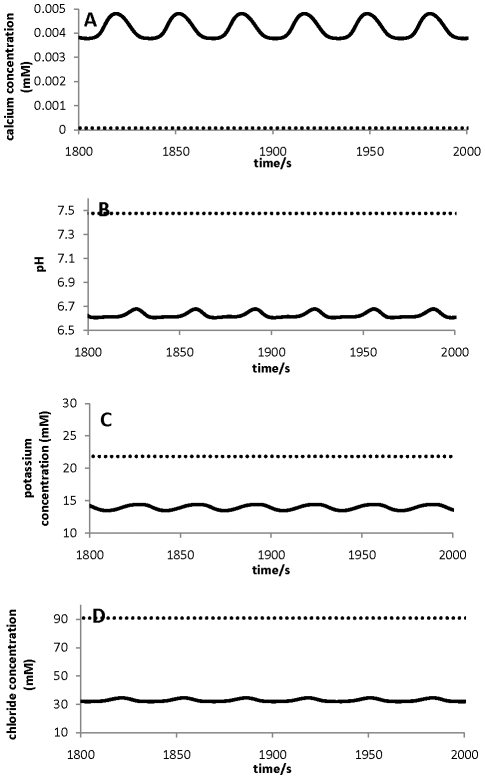
Ion gradients. Ion gradients are established between the tip and shank for four major ions. All ions at the tip (solid line) are with oscillatory dynamics; and all ions at the shank (dashed line) are with non-oscillatory dynamics.

Essential features for pollen tube growth include the polarisation of ion fluxes, intracellular ion gradients, and oscillating ion and growth dynamics [11], [12]. The tip-shank two-compartment model (figure 1) with the parameter values in Tables 1 and 2 generates different intracellular ion concentrations at the tip and shank, oscillatory ion dynamics at the tip, and non-oscillatory ion dynamics at the shank. For the convenience of comparing modelling results with experimental observations, throughout this work we refer “different intracellular ion concentrations at the tip and shank” in the two-compartment model as “ion gradients between the tip and shank”. The results are summarised in figure 2 for all four ions Ca^2+^(figure 2A) pH (figure 2B), K^+^ (figure 2C) and Cl^−^ (figure 2D).

**Table 1:** Parameters for all 12 transporters in Figure 1.

Transporter	Parameter
t1: inward rectifying K^+^ channel	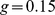 S m^−2^ mM^−1^, 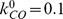 s^−1^, 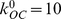 s^−1^
t2: outward rectifying K^+^ channel	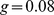 S m^−2^ mM^−1^, 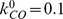 s^−1^, 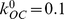 s^−1^
t3: Ca^2+^ channel	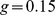 S m^−2^ mM^−1^, 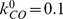 s^−1^, 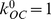 s^−1^
t4: H^+^ ATPase pump	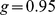 S m^−2^, 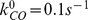 , 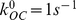
t5: Cl^−^-2H+ symporter	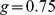 S m^−2^, 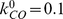 s^−1^, 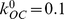 s^−1^
t6: Cl^−^ channel	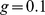 S m^−2^ mM^−1^, 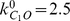 s^−1^, 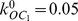 s^−1^, 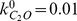 s^−1^, 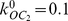 s^−1^
s1: inward rectifying K^+^ channel	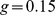 S m^−2^ mM^−1^, 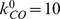 s^−1^, 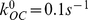
s2: Ca^2+^ channel	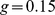 S m^−2^ mM^−1^, 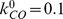 s^−1^, 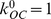 s^−1^
s3: Ca^2+^ ATPase pump	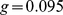 S m^−2^, 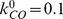 s^−1^, 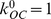 s^−1^
s4: H^+^ ATPase pump	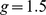 S m^−2^, 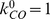 s^−1^, 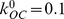 s^−1^
s5: Cl^−^-2H+ symporter	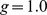 S m^−2^, 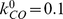 s^−1^, 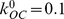 s^−1^
s6: Cl^−^ channel	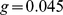 S m^−2^ mM^−1^, 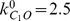 s^−1^, 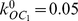 s^−1^, 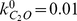 s^−1^, 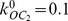 s^−1^

**Table 2:** Constants and other parameters.

description	parameter
Faraday constant: F	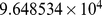 C mol^−1^
Boltzmann constant: 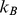	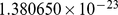 JK^−1^
elementary charge: e	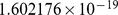 C
Gas constant: 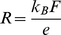	8.3145 J K^−1^mol^−1^
Temperature: T	T = 273.15+25 = 298.15 K
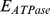 : Reversal potential for ATPase contribution in all pumps. It is the same at both tip and shank	−0.45V [7], [8].
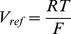	0.02569 V
Rate constants for ion exchange between both tip and shank	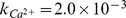 s^−1^, 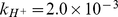 s^−1^, 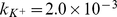 s^−1^, 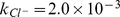 s^−1^
Volume at both tip and shank	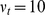 µm^3^, 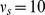 µm^3^
Surface that is occupied by transporters to volume ratio at both tip and shank are the same	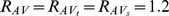 µm^−1^
The volume ratio between tip and shank: 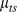	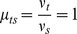
Membrane capacitance at tip and shank	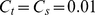 Fm^−2^

In figure 2A, the calcium concentration is higher at the tip than that at the shank and the calcium concentration oscillates between 0.0038mM and 0.0048 mM with an amplitude of approximately 0.001mM at the tip and does not oscillate at the shank. At the shank the steady-state concentration is *ca.* 7.5×10^−5^ mM. To be precise, when oscillations emerge at the tip, the “steady state” at the shank is an oscillatory state but with an amplitude of <7.0×10^−9^ mM and it is anticipated that such a small-amplitude oscillation would not be detected using current experimental procedures. As a result we will be referring this oscillatory state to be a “steady state”, in order to maintain agreement with the terminology of experimental observations in pollen tube growth. However, mathematically, this “steady state” is different from the steady state which is established when oscillations do not emerge at the tip. For the latter case, both tip and shank establish a steady state without oscillations. The pH at the tip is lower than that at the shank (figure 2B). Again, the pH value at the tip oscillates and this is between 6.6 and 6.7, and the pH value at the shank does not oscillate (∼7.47) (figure 2B). These results are in close agreement with experimental observations in lily and tobacco pollen tubes [5], [11], [12], [24]. We note that, while the calcium dynamics in pollen tubes have been extensively studied [11], [12], the measurements of pH dynamics in pollen tubes are relatively rare [5], [24]. In addition, both potassium and chloride also establish gradients between the tip and shank. Both potassium and chloride concentrations at the tip are lower than at the shank (figures 2C and 2D). To our knowledge, there are no accurate experimental data available for intracellular potassium and chloride concentrations, although fluxes of potassium and chloride were experimentally measured [11], [12], [16], [21], [22], [23].

### Polarised ion fluxes

**Figure 3:**
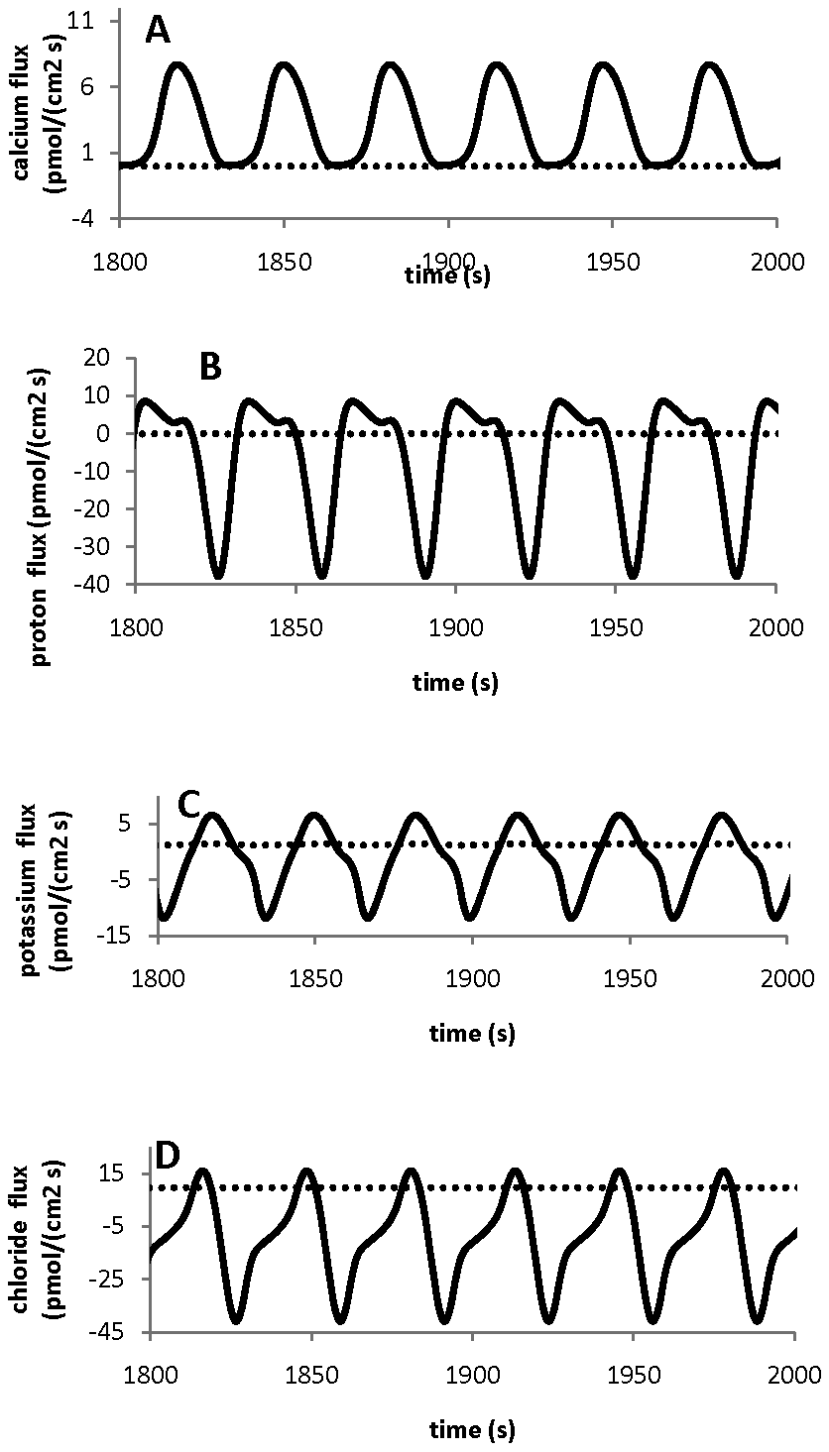
Ion fluxes. Polarised ion fluxes at the tip (solid line) and shank(dashed line) compartments in a pollen tube.

As shown in Figure 3, ion gradients between the tip and shank, Figure 2, are generated by polarised ion fluxes, which are oscillatory at the tip, and non-oscillatory at the shank.

In figure 3A, it is shown that there is an oscillatory calcium influx at the tip and a steady-state efflux at the shank (−0.02 pmol/(cm^2^s)). This result agrees well with experimental observations [11], [12]. For H^+^, there is an alternative oscillatory influx and efflux at the tip and an non-oscillatory efflux at the shank (−0.1 pmol/(cm^2^s)) (figure 3B). Experimentally, it was reported [11] that there is an non-oscillatory efflux of −0.4 pmol/(cm^2^s) at the shank, and an oscillatory influx at the tip (0–0.4 pmol/(cm^2^s)). Figure 3C shows that potassium comes out and goes in the tip with an oscillatory flux at the tip. At the shank, potassium goes in with a non-oscillatory flux of 1.4 pmol/(cm^2^ s). Although experimental measurements for potassium in pollen tube are debatable [11], [12], it seems that a consensus is that potassium goes in from shank with a non-oscillatory flux and it may go in and come out at tip [11], [12], [23]. Therefore, the modelling results of potassium fluxes qualitatively agree with those of experimental observations. Figure 3D shows that chloride goes in at the shank with a non-oscillatory influx of 9.6 pmol/(cm^2^s), and goes in and comes out at the tip with an oscillatory flux. Although there is only limited knowledge about chloride fluxes, it seems that chloride goes in at the shank with a non-oscillatory flux and comes out at the tip with an oscillatory flux [11], [12], [16], [21], [22]. Therefore, the modelling results for chloride qualitatively agree with experimental observations. Quantitatively, we note that some much larger fluxes for both potassium and chloride were reported in literature [11], [16] and experimental measurements for potassium and chloride in pollen tube are subjected to debating [16].

**Figure 4:**
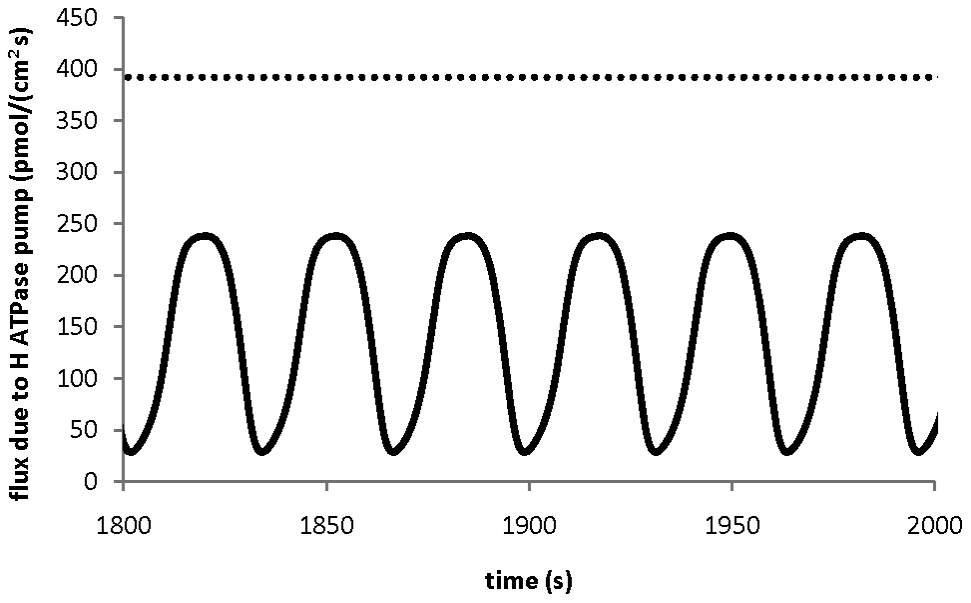
Fluxes due to H+ ATPase pump. Comparison of fluxes due to H+ ATPase pump at the tip (solid line) and at the shank (dashed line).

Based on the model analysis, the localised transporters at the tip and shank are able to generate the polarised fluxes (figure 3), which result in ion gradients between the tip and shank (figure 2). In addition, figure 4 shows the fluxes of H^+^ due solely to the H^+^ ATPase pump at both the tip and shank. It reveals that the average flux at the tip is much lower and it is ∼25% of that at the shank. Experimentally, it was shown [15] that the abundance of H^+^ ATPase transporter at the tip is much lower than that at the shank. Although the quantitative relationship between the abundance of H^+^ ATPase transporter and the fluxes generated by H^+^ ATPase transporter is unknown, it is reasonable to assume that lower abundance corresponds to lower fluxes with an unknown nonlinear relationship. In general, the abundance (or concentration) of a protein is proportional to the flux that the protein can generate although other effectors may also regulate the flux [25]. The relationship between the abundance (or concentration) of a protein and the flux can be generally described by mass-action kinetics or Michaelis-Menten kinetics [25]. Therefore, the developed model qualitatively agrees with experimental observations on the localised distribution of H^+^ ATPase pump and it has incorporated the difference of H^+^ ATPase transporter abundance between tip and shank.

### Varying external ion concentrations

**Figure 5:**
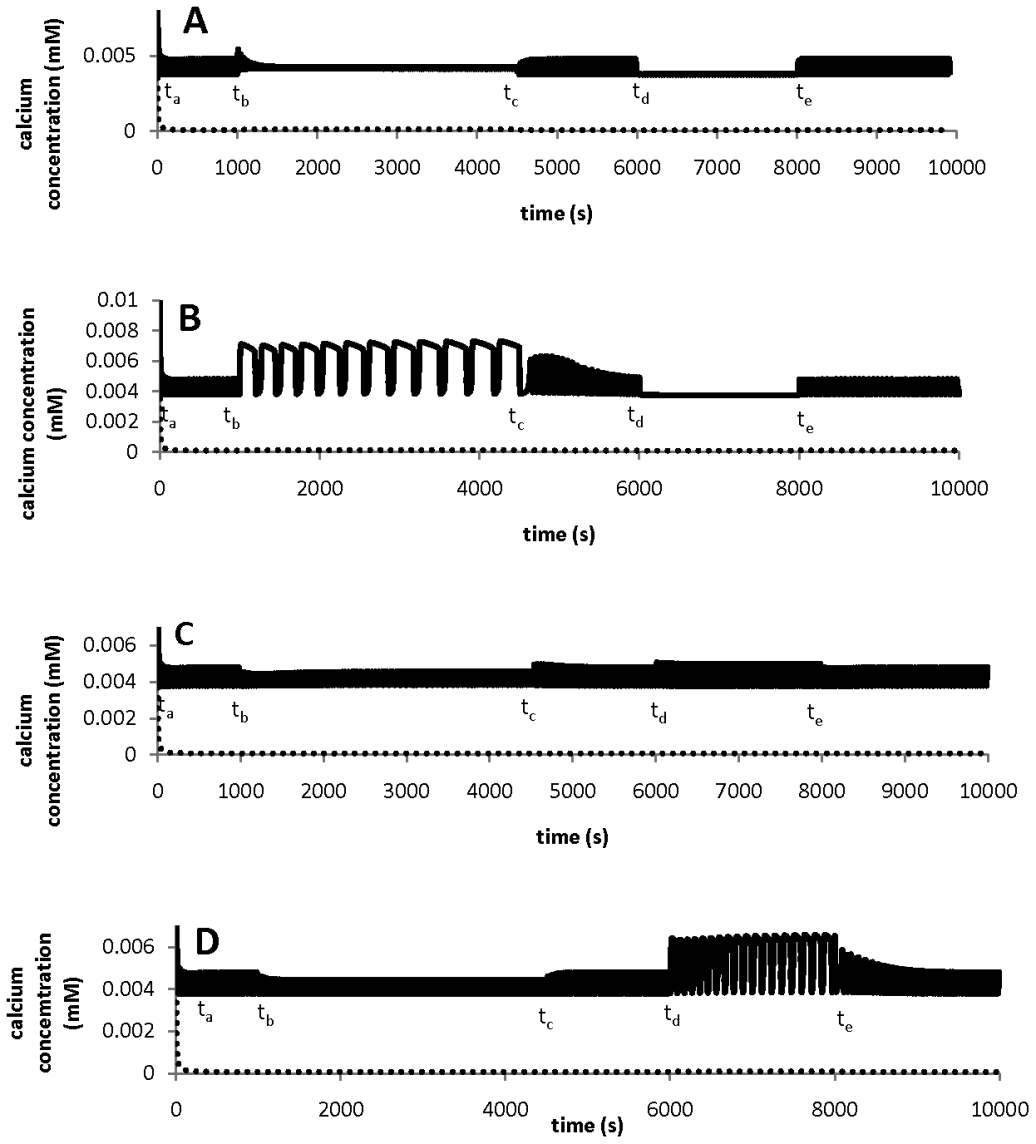
Response of calcium concentration to changes in external ion concentrations. The tip : solid line; the shank: dashed line. These responses qualitatively agree well with experimental observations [26]. A: changes in external calcium concentration: t_a_: [Ca]_e_ = 1mM; t_b_: [Ca]_e_ = 5 mM; t_c_: [Ca]_e_ = 1 mM; t_d_: [Ca]_e_ = 0.05 mM; t_e_: [Ca]_e_ = 1 mM. B: changes in external pH: t_a_: pH_e_ = 5.5; t_b_: pH_e_ = 6.5; t_c_: pH_e_ = 5.5; t_d_: pH_e_ = 4.5; t_e_: pH_e_ = 5.5. C: changes in external potassium concentration: t_a_: [K]_e_ = 1mM; t_b_: [K]_e_ = 3 mM; t_c_: [K]_e_ = 1 mM; t_d_: [K]_e_ = 0.03 mM; t_e_: [K]_e_ = 1 mM. D: changes in external chloride concentration: t_a_: [Cl]_e_ = 1mM; t_b_: [Cl]_e_ = 1.5 mM; t_c_: [Cl]_e_ = 1 mM; t_d_: [Cl]_e_ = 0.015 mM; t_e_: [K]_e_ = 1 mM.

For all calculations, we set the external medium composition 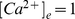 mM; pH_e_ 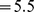; 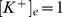 mM; and 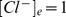 mM as a reference point. Figure 5 summarises the results for varying one of four external ion concentrations each time.

Figure 5 shows that when 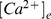, 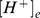, 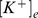 or 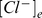 varies 100 fold, the baseline of intracellular calcium concentration does not significantly change, implying that pollen tube system is capable of maintaining a stable level of intracellular calcium concentration when ion concentrations fluctuate, as observed experimentally in lily pollen tubes [26]. However, as shown in figure 5, oscillatory dynamics at the tip may depend on the concentrations of ions in the medium, as discussed below. When 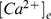 is increased from 1 mM to 5 mM, the oscillatory amplitude is reduced and further increase in 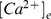 eliminates the oscillation, Figure 5A. In addition, model simulations show that as long as oscillations exist, changing 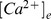 does not markedly affect oscillating frequency. When 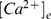 changes back to the reference value 1 mM, the original oscillatory state is always recovered. These results qualitatively agree with experimental observations [26]. Experimentally, 0.13 mM 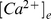 was used as a reference point [26]. It was observed that increasing 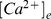 to 1.3 mM results in small-amplitude oscillation and increasing 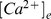 to 10 mM approximately eliminates oscillations. Although experimental data were noisy, it seems that the oscillating frequency is not significantly affected by changing 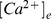 [26]. Model simulations also show that if 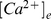 is reduced to 0.05 mM from its reference value 1mM, then the oscillating amplitude is reduced. Figure 5B shows that when pH_e_ is increased from 5.5 to 6.5, large-amplitude and low-frequency oscillations are formed. However, when pH_e_ is decreased from 5.5 to 4.5, the oscillations are eliminated. These results qualitatively agree with experimental observations. Experimentally, at pH_e_ = 6.5, irregular large-amplitude and low-frequency oscillations are observed [26]. However, at pH_e_ = 4.5, small-amplitude noisy oscillations are found [26]. Figure 5C shows that 100-fold change of 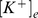 significantly affects neither the oscillatory frequency nor the amplitude. Numerical test shows that when 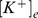 is reduced to as low as 0.01 mM, the oscillations remain. However, if 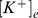 is increased to 4mM or more, the oscillations disappear. These results qualitatively agree with experimental data [26], although the oscillations are experimentally observable for a wider range of 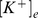 (0.01mM–10mM). Figure 5D shows the effects of changing 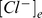. Increasing 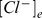 decreases the oscillatory amplitude. When 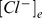
>2 mM, the oscillations disappears. Reducing 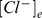 may generate large-amplitude and low-frequency oscillations. To our knowledge, there are no experimental results about varying 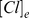 for comparison.

Our modelling results are in good agreement with experimental observations a) that ion gradients between the tip and shank are established, with a feature of oscillating dynamics at the tip and a steady state at the shank; b) that ion gradients between the tip and shank are due to polarised ion fluxes [11]; c) that the abundance of H^+^ ATPase transporters at the tip is much lower than that at shank [15]; d) that the period and amplitude of calcium oscillations respond qualitatively to different external ion concentrations (calcium, potassium and pH [26]). It is reasonable to suggest that the two-compartment model that we present has captured the main features of the interactions between the tip and the shank in a pollen tube. Therefore, we have used the model in the following sections to further analyse ion and growth dynamics in the pollen tube.

### Integrative ion dynamics for responding to perturbations

When a pollen tube is subjected to any perturbation, how does the pollen tube develop its response? Here, we subject a pollen tube to different perturbations and investigate the underlying mechanism for the response.

**Figure 6:**
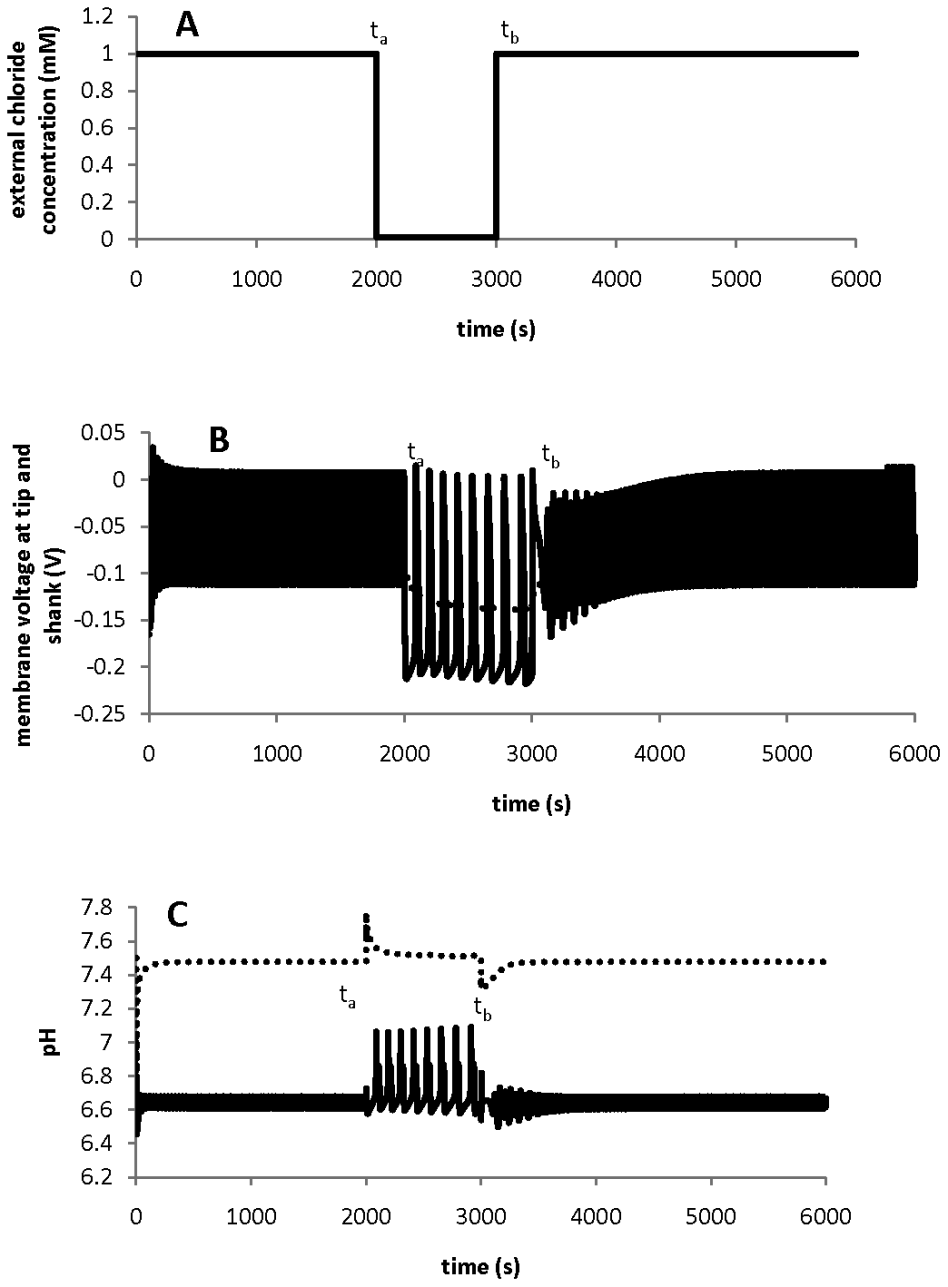
Integrative response of the tip and shank. When external chloride concentration is perturbed, the integrative response of the tip (solid line) and shank (dashed line). A: perturbation of external chloride concentration. B: the response of membrane voltage at both the tip and shank; C: pH response at both the tip and shank. t_a_: perturbation is on; t_b_: perturbation is off.

Figure 6 shows how a pollen tube responds to perturbation by external chloride concentration. When the external chloride concentration is reduced to 0.01mM at 2000s and recovered to the original level (1 mM) at 3000 s (figure 6A), the currents for chloride transporters at both tip and shank (t5, t6, s5, s6 in figure 1) change immediately. Such changes in the currents induce changes in membrane voltages at both the tip and the shank (figure 6B). Once membrane voltages change, they act as “global regulators” regulating the kinetic properties of all channels and pumps at both the tip and shank. Consequently, the currents relating to all transporters change. Following equations (1) and (2), changes in the currents lead to changes in the four major ion concentrations and figure 6C shows the change in pH. In turn, changes in the ion concentrations again contribute to changes in currents and voltages a) by changing the currents through the membrane at both the tip and shank and b) by changing ion gradients that lead to the changes in the current travelling along a pollen tube. Therefore, following the perturbation by external chloride concentration, the tip and shank respond as an integrative dynamical system, resulting in a coordinated response. This analysis is applicable to perturbations by any external ions. When the imposed external perturbation is removed, the integrative dynamics re-establish their original ion gradients. Moreover, perturbations to the intracellular ion concentrations can be examined by adding a term in equation (1) or (2) for either producing an ion or consuming an ion. This kind of perturbation is biologically equivalent to the perturbations introduced by intracellular biological processes. Although internal and external perturbations may be realised using different biological means, the response of the pollen tube to internal perturbations follows the same underlying mechanism as its response to external perturbations. A detailed analysis on internal perturbations and on the insensitivity of ion dynamics to initial conditions is included in the Data S2 in the Supporting Information.

Based on the analysis above, we can conclude that the four major ions at the tip and the shank of a pollen tube form an integrative dynamical system which is insensitive to the initial setup of ion concentrations. Therefore, when any perturbation experiment is designed or when such experimental data are explained, this integrative response strategy should be taken into account.

### Oscillatory dynamics at the tip are not important for establishing ion gradients

**Figure 7:**
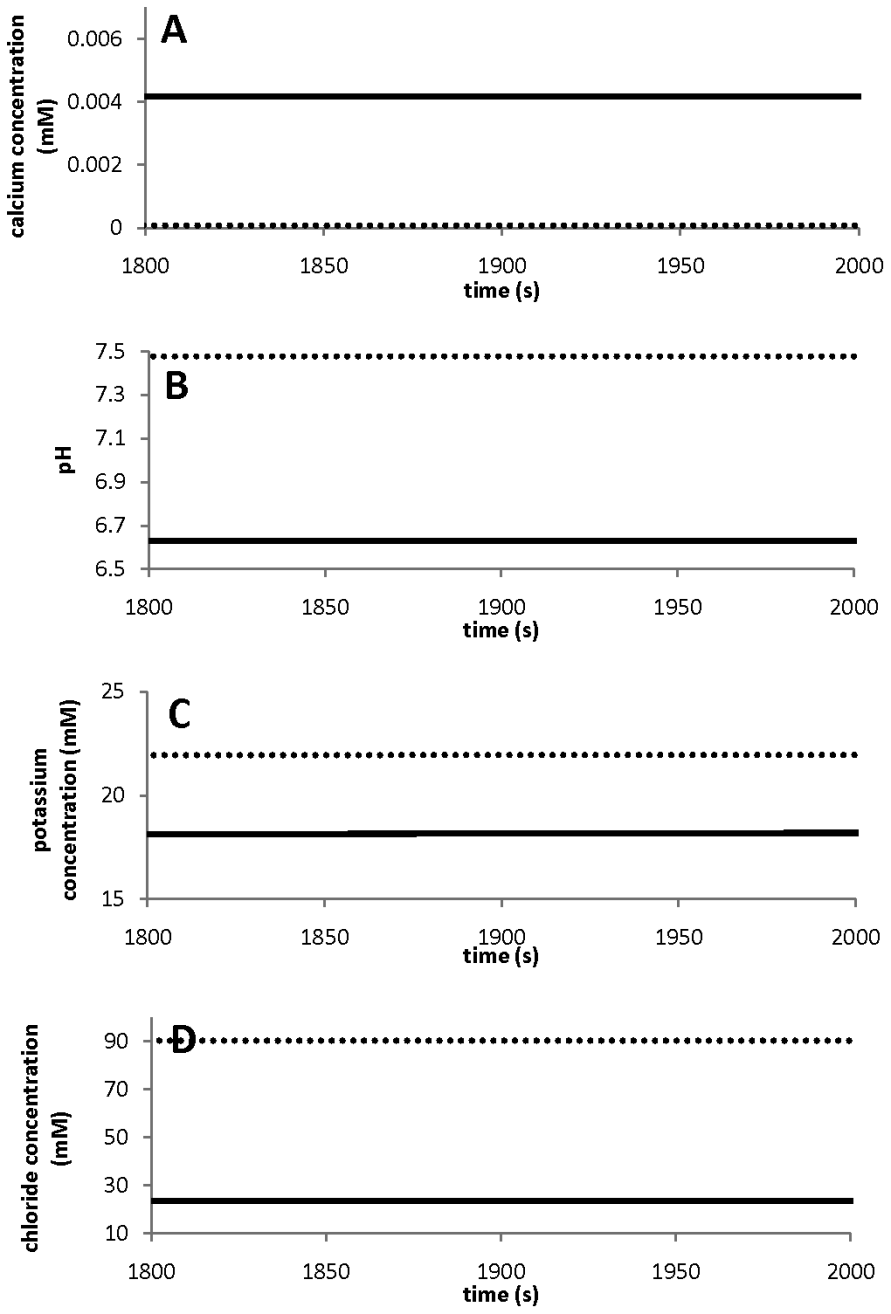
Ion gradients with non-oscillatory dynamics. An example for the formation of ion gradients for all four major ions with non-oscillatory dynamics at both the tip (solid line) and shank (dashed line). 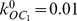
s^−1^ at the tip and all other parameters are the same as in Tables 1 and 2.

A striking feature of ion dynamics in the pollen tube is the oscillatory dynamics at the tip and the non-oscillatory dynamics at the shank both of which coexist with intracellular ion gradients. An important question is whether oscillatory dynamics are important for the formation of ion gradients. Therefore, we have further examined the factors that affect the formation of ion gradients. By changing the kinetic parameters shown in Tables 1 and 2, oscillations at the tip can be generated or eliminated. However, using the model we find that ion gradients can be maintained, independently of the emergence of oscillatory dynamics. An example for the formation of ion gradients with non-oscillatory dynamics at both tip and shank is included in figure 7. Therefore, oscillatory dynamics at the tip may be the consequence of the integrative dynamical system rather than the requirement to form ion gradients between the tip and the shank. However, we note that when oscillatory dynamics exist, ion concentrations and fluxes change in a range over time. Whether or not these oscillations in ion concentration have biological significance cannot be addressed by this theoretical model.

### Role of a net current travelling along pollen tube

Experimentally, it has been shown that there is a net current entering from the tip and leaving by the shank of the lily pollen tube [14]. What is the role of such a current? Using the two-compartment model, we suggest that the current stems from the summation of all four ion gradients across the tip and shank (equation (e12) in Materials and Methods). Once the current travelling along a pollen tube changes i.e. by changing the ion gradients, the membrane voltage at both the tip and shank adjust accordingly in accordance with equations (e13) and (e14) in Materials and Methods. Subsequently, ion concentrations at both the tip and shank adjust and new ion gradients are formed. Therefore, regulation of ion gradients effecting membrane voltage generates a self-regulating loop for ion concentrations at both the tip and shank i.e. changes in ion concentrations at either/both the tip and shank will lead to changes in ion gradients that, in turn, will alter the ion concentrations at both the tip and shank.

Our numerical studies also reveal that by manipulating the current travelling along a pollen tube by changing some parameters (e.g. diffusion coefficient or kinetic parameters), one can alter the properties of ion dynamics. For example, when one of the kinetic parameters of the Cl^−^ channel at the tip is changed, the oscillations at the tip can disappear (Figure 7). Subsequently, although both the tip and shank have steady-state ion concentrations, ion gradients can be maintained (Figure 7).

### Mechanisms for growth-induced oscillations

The growth rate of a pollen tube is controlled by many factors including ion concentrations. In this context it is reasonable to assume that the growth rate is related to all four major ions, but the quantitative relationship between the ion concentrations and growth rate are largely unknown. Therefore, in this work, we propose to use a power-law formalism to describe the relationship between ion concentrations and growth rate. Power-law formalism or S-system theory was developed by Savageau et. al [27], [28] and it is a useful methodology to describe complex biological interactions, in particular when detailed kinetic knowledge of the underlying biological processes is unknown [27], [28]. Different types of kinetic equations can be re-casted into power-law formalism [28]. Following the power-law formalism, the growth rate can be generally expressed as
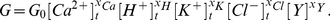
(3)In equation (3), 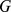 is the growth rate, and 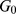 is a constant. 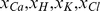 and 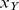 are the powers for the four ions at the tip and an unknown factor Y, and these powers can be negative, positive or zero. All concentrations are in mM. Using equation (3), the growth rate is coupled with ion dynamics. Since the quantitative relationship between growth rate and ion concentrations is largely unknown, for simplicity, we take 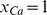, 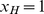, 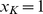, 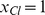 and 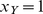. When 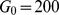
µm^3^/(s mM^5^) the result is a growth rate of ∼0.1 µm^3^/s where [Y] = 1 mM and 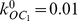
s^−1^ for the chloride channel at the tip and all other parameters are as in Tables 1 and 2. We note that the growth rate described in this work represents volume change.

We have incorporated the growth rate into our two-compartment model and study the effects of growth rate in terms of the incorporation of new membrane at the tip in relation to ion dynamics. As new membrane is formed at the tip, the tip volume increases. However in order for the tip and shank volumes to remain constant the tip volume must be converted into shank volume. For an oscillatory growth rate, the simplest rule for accommodating both the incorporation of new membranes at the tip and an approximately constant tip and shank volume, as the pollen tube grows, is as follows. a) the total volume of tip and shank is constant; (b) the tip volume is converted to shank volume after a delay time, 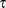. Therefore, following this rule, the tip and shank volumes can be described using equation (4).
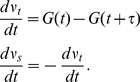
(4)where 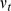 and 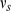 are the volume for the tip and shank compartment, respectively. Following equation (4), in equations (1) and (2), 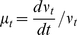; 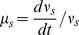 and 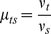. After coupling equations (3) and (4) with equations (1) and (2), the third term in both equations (1) and (2) is non-zero and it describes the effects of volume changes on ion concentrations. As the total volume of tip and shank is constant following equation (4), it implies that the shank volume is also converted into a third compartment (body volume) with a rate of 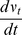. If the body volume is included in the model, an extra term in equation (2) should be included to describe the exchange of ions between the shank and body in the form of the second term of equation (2), if the ion concentrations in the shank and body compartments are different (see the Data S1 in the Supporting Information for details). We have numerically tested the effects of the body compartment, finding that inclusion of the body compartment does not affect our conclusions. Therefore, for simplicity, we use equations (1–4) to fully describe the mass balance when the growth rate is incorporated into ion dynamics. An implicit assumption for equations (1–4) is that the concentration of four major ions in the shank compartment is the same as its counterpart in the body compartment (see the Data S1 in the Supporting Information for details).

Using equations (1–4), we have now established a fully coupled system which integrates growth rate with ion dynamics. In the following section, we will examine the interplay between ion dynamics and growth rate focussing on the mechanism for generating the oscillations.

Clearly, as ion dynamics generate oscillations, the growth rate will have to oscillate also according to equation (3). However, if ion dynamics do not oscillate, we wanted to determine whether any oscillations in the growth rate can induce oscillations in ion concentrations and ion fluxes. To address this point, we set up our two compartment model as follows. Firstly, we change the value of one parameter in Table 2 (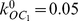
s^−1^ to 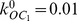
s^−1^ for the chloride channel at the tip) so that the oscillations in ion dynamics are eliminated. Secondly, we introduced sinusoidal oscillations in the growth rate by changing [Y] = 1mM into
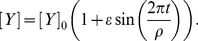
(5)In equation (5), 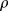 and ε are the period and amplitude for oscillations in [Y], respectively, and 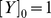 mM. Equation (5) indicates that there is an unknown source (any source other than ion concentrations) generating oscillations with a period, 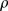, and an amplitude, ε, in growth rate. We note that the average [Y] over an exact period of 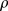 is 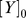, and this is still 1 mM, indicating that equation (5) solely introduces the effects of oscillations rather than the effects of changing the average [Y].

**Figure 8:**
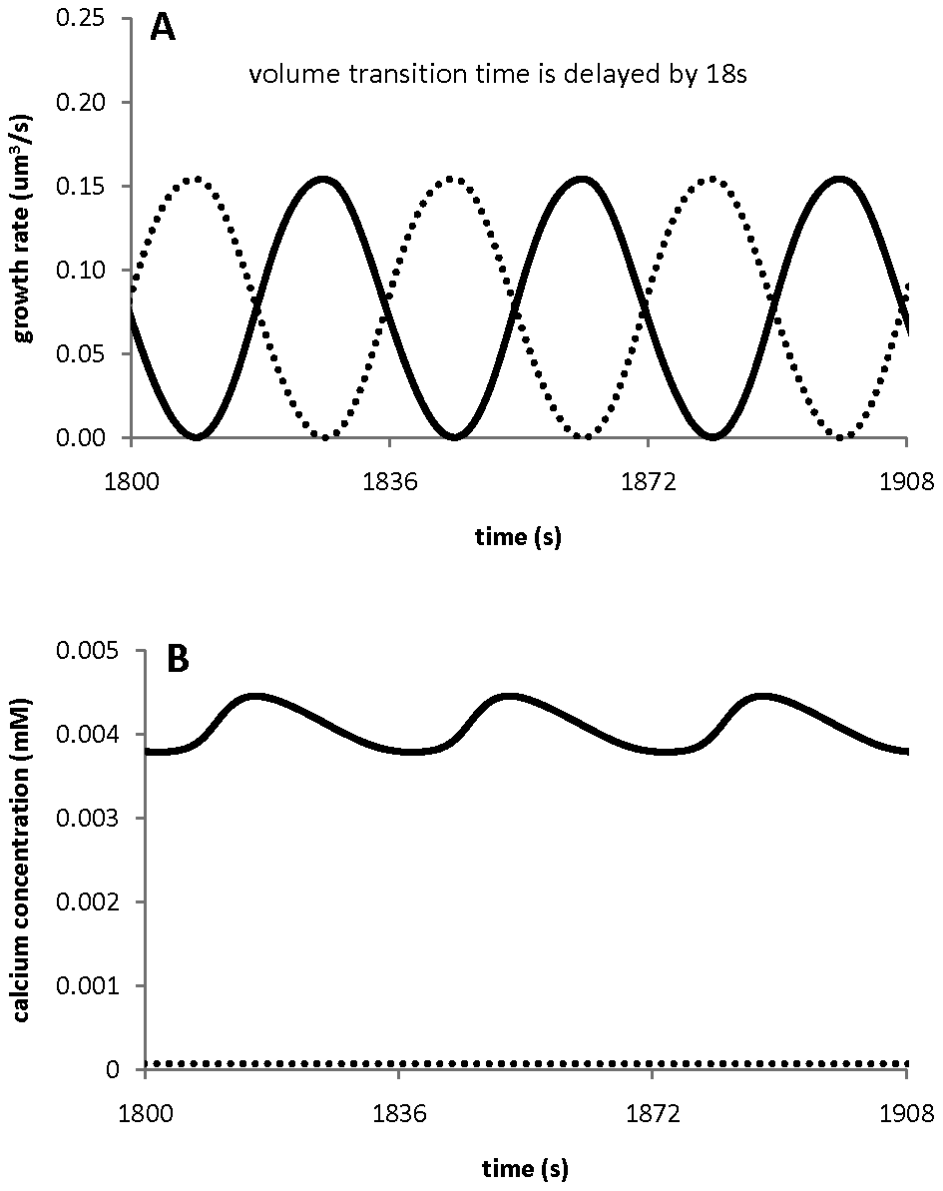
Oscillations induced by the volume transition. Oscillations at the tip can be induced by the transition of the tip volume to the shank volume. A. transition of tip volume to shank volume is delayed by 18s when [Y] periodically changes following equation (5) with 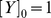
mM, 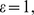 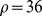
s. B. Growth-induced oscillations in calcium concentration at the tip. 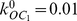
s^−1^ for chloride channel at the tip and all other parameters are the same as in Tables 1 and 2. Tip: Solid line; shank: dashed line.

Our numerical studies reveal that when oscillations exist in the growth rate via an unknown factor [Y] (equation (5)), two mechanisms for generating oscillations in ion concentrations and fluxes can be proposed:

The driver for the first mechanism is the transition from tip volume to shank volume. Figure 8 summarises the results.

When 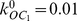 s^−1^ for the chloride channel at the tip and when all other parameters are taken as being the same as in Tables 1 and 2, the system becomes settled into steady state at both the tip and shank where [Y] = 1mM. When the growth rate oscillates as a result of the periodic changes in [Y] where 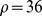
s^−1^ and 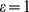 the tip volume will increase due to the addition of new membrane at the tip but it will decrease due to the transition of tip membrane to the shank membrane and the phase shift between both events is 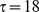
s, (figure 8A). As a direct result of this transition between tip volume and shank volume, all ion concentrations and fluxes at the tip will oscillate but the tip and shank volumes will remain unchanged (figure 8B). These oscillations are similar to the intrinsic oscillations generated by ion dynamics that are shown in figure 2. The period of this type of growth-induced oscillation in both ion concentrations and fluxes is the same period as that of the growth rate itself. Consequently for this type of mechanism, if 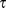 = 0 s or 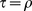 s, then no oscillations will be induced because both the addition of new membrane and the conversion to shank membrane will be in phase. The driver for the second mechanism relies on the periodic changes in some of the kinetic parameters that are induced by the oscillations in the growth rate. For example, if it is assumed that 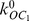 for the chloride channel at the tip becomes 
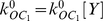
 when [Y] oscillates following equation (5) (note that the average 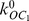 for the chloride channel at the tip is still 0.01 and with a unit of s^−1^mM^−1^ as the average of [Y] is 1mM), oscillations in ion concentrations and fluxes will be generated and these are qualitatively similar to those oscillations described in figure 8B. This mechanism requires that periodic oscillations in the growth rate periodically change some kinetic properties. For example, if the probability of an ion transporter to be open relates to the abundance of this transporter in the membrane then the abundance of the transporter should change periodically.

### Integrative and self-regulatory ion dynamics in pollen tube

**Figure 9:**
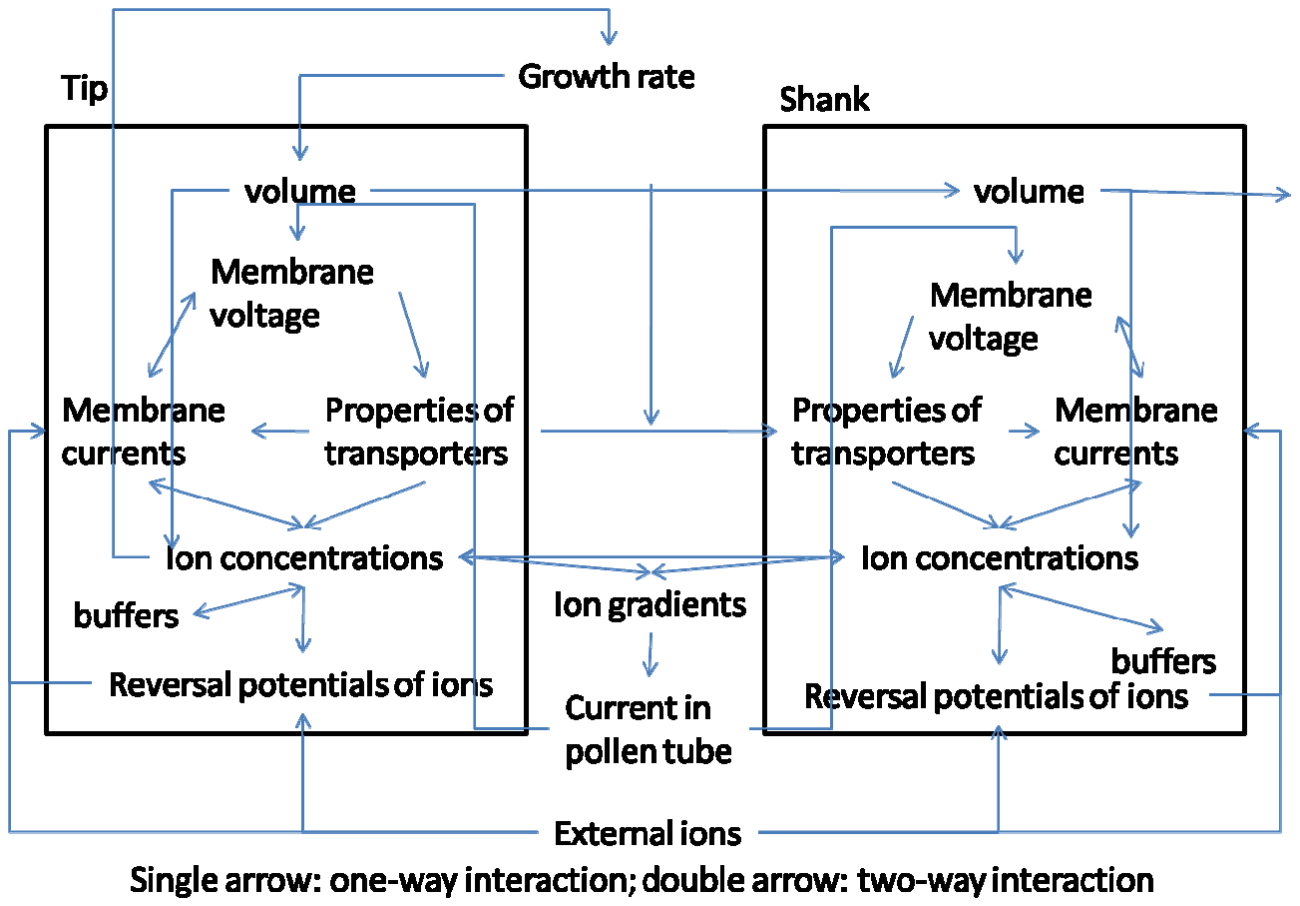
Integrative and self-regulatory ion dynamics revealed and analysed by the two-compartment model (**Figure 1**).

The establishment of a two-compartment model has revealed that the tip-shank interaction in a pollen tube forms an integrative and self-regulatory ion dynamic network, as summarised in Figure 9. When any link within the network is perturbed, the system responds in an integrative and self-regulatory way and the effects are transmitted to both tip and shank even though the tip and shank have different transporters. If, for example, the growth rate is modified by changes in actin dynamics [29], a volume change induced at the tip will subsequently lead to changes in ion concentrations within the network which will also feedback into the regulation of the growth rate. Similarly, if a current travelling along a pollen tube is modified, for example by changing the ion concentrations at either the tip or the shank, then the membrane voltage at both tip and shank will be effected which will lead to a change in ion concentrations which will feedback and regulate the current travelling along the pollen tube. The two-compartment model developed in this work is able to quantify how the pollen tube system responds to any perturbation in this integrative and self-regulatory manner. Moreover, as described in Data S3 in the Supporting Information, the two compartmental model can be abstracted into an electrical dipole circuit. This model will now act as a platform for the addition of further regulatory modules in future work.

### Relationship between transporters and ion dynamics

**Figure 10:**
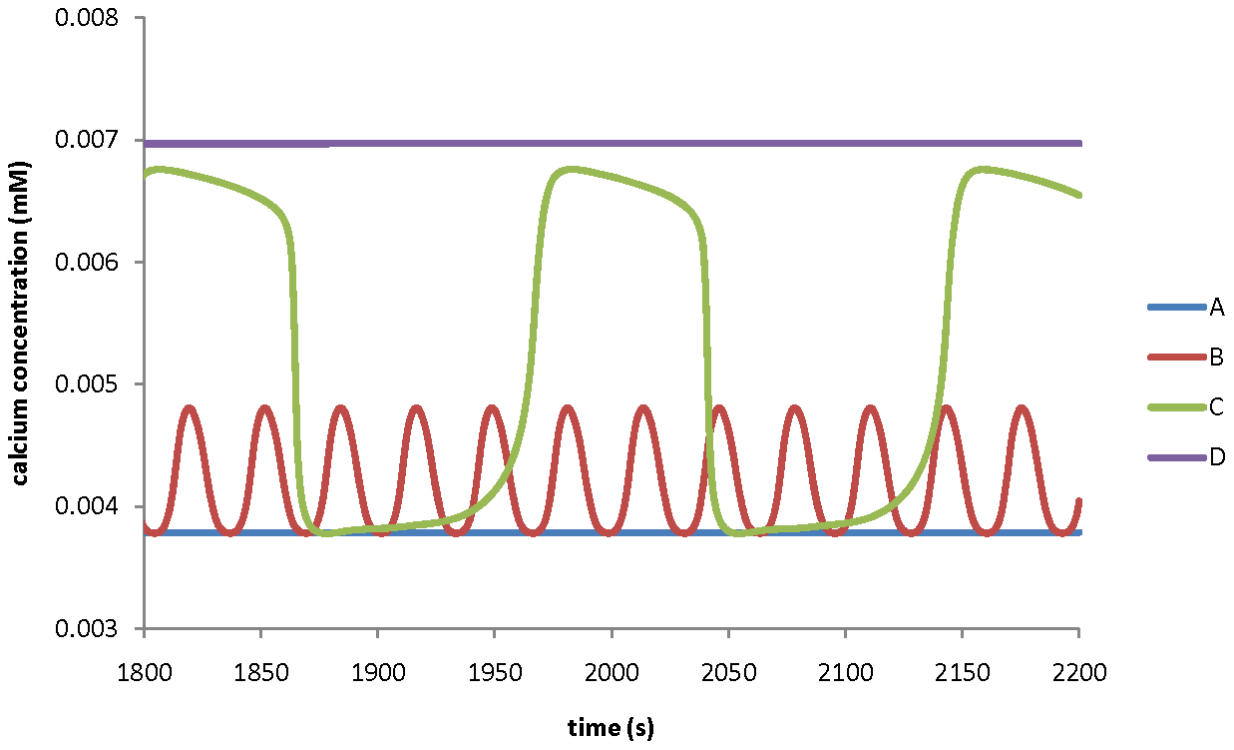
Modelling prediction for the effects of the maximum conductance of H^+^ ATPase pump on calcium ion dynamics at the tip. The maximum conductance of H^+^ ATPase pump at the tip is A) 0.8 S m^−2^ ; B) 0.95 S m^−2^; C) 1.5 S m^−2^; D) 2.5 S m^−2^ respectively. All other parameters are the same as in Tables 1 and 2.

The model developed in this work is able to predict how manipulation of any transporter affects ion dynamics in a pollen tube. Figure 10 shows the effects of H^+^ ATPase pump on calcium ion dynamics at the tip.

Figure 10 shows that if the maximum conductance of the H^+^ ATPase pump is reduced from 0.95 S m^−2^ to 0.8 S m^−2^, the oscillations in calcium concentration at the tip are eliminated and calcium concentration reaches a steady state with a lower value (∼0.0038 mM). However, if it is increased from 0.95 S m^−2^ to 1.5 S m^−2^, the oscillations in calcium concentration increase in both period and amplitude. Moreover, if the maximum conductance of the H^+^ ATPase pump is further increased to 2.5S m^−2^, the calcium concentration reaches a steady state with a higher calcium concentration (∼0.007 mM). Therefore, changes in the kinetic property of the H^+^ ATPase pump may change the dynamics of calcium ions. In contrast, a 5-order change in the maximum conductance of either K^+^ inward or outward rectifying channels at the tip (0.0001–10 S m^−2^ mM^−1^) does not eliminate the oscillations in calcium concentration. Similarly, the effects of other transporters on ion dynamics have been examined. It has been predicted that the oscillatory ion dynamics at the tip respond sensitively to the changes in the values of the kinetic parameters for the following transporters: H^+^ ATPase pump, Cl^−^-2H^+^symporter and chloride channel at the tip. However, the oscillatory dynamics at the tip will stay within a wide range of kinetic parameter values for the following transporters: K^+^ inward and outward rectifying channels, calcium channels at the tip. Model analysis also shows that the oscillatory dynamics at the tip are insensitive to the kinetic parameters at the shank.

Molecular data on the relevant transporters in pollen tubes have been experimentally determined in *Arabidopsis* [12], [13]. Following the predictions of our model, either reducing or enhancing the expression level of the H^+^ ATPase pump, Cl^−^-2H^+^symporter or chloride channel may either significantly change or eliminate the oscillatory dynamics at the tip during pollen tube growth. Further experimental measurements where the expression levels of the H^+^ ATPase pump, Cl^−^-2H^+^symporter or chloride channel is manipulated may help in elucidating the mechanism that underlies the oscillatory dynamics at the tip in pollen tube growth.

### Effects of volume and growth rate on the ion dynamics

If the volume or the growth rate at the tip does not affect the surface (that is the surface occupied by transporters) to volume ratio, the oscillatory dynamics do not markedly change with the volume or growth rate. This implies that when the volume of a pollen tube increases, the oscillatory dynamics can maintain if the surface also proportionally increases. However, if the volume or the growth rate changes affect the surface to volume ratio, the oscillations may change their period and amplitude.

**Figure 11:**
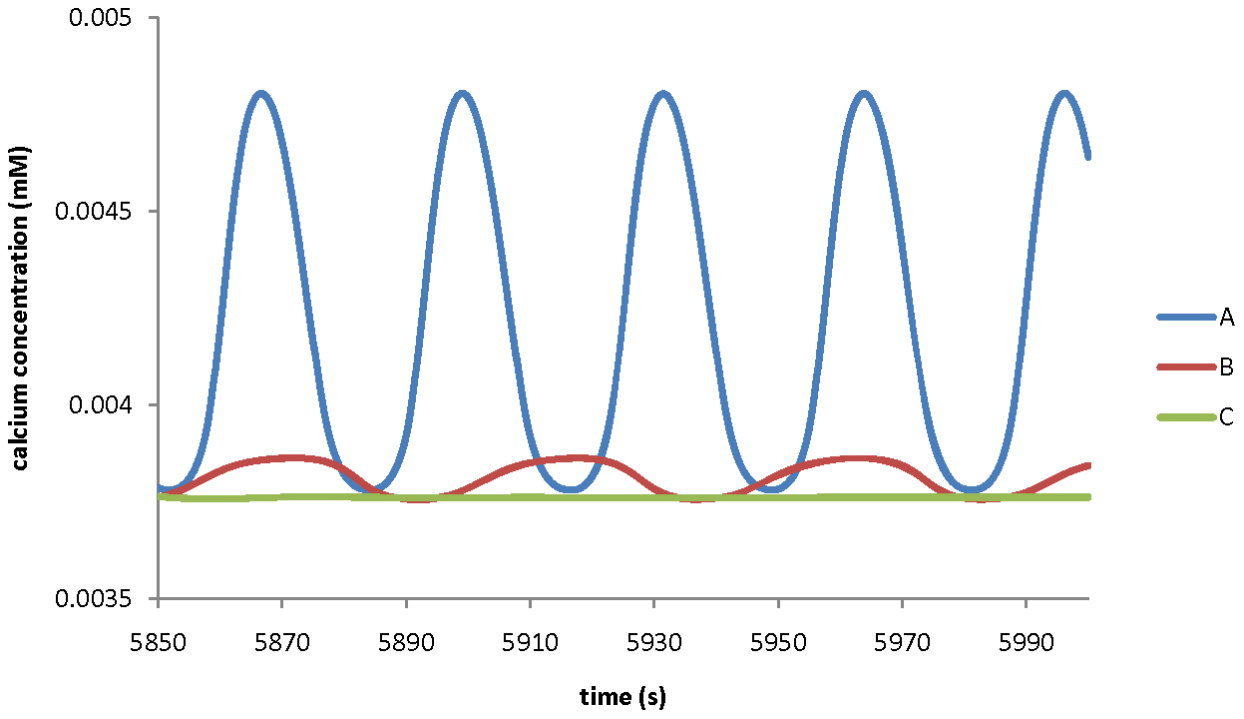
Effects of growth rate on the dynamics of calcium concentration. The tip volume increases following growth rate. The surface that is occupied by transporters to the volume ratio decreases following 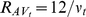
µm^−1^. All other parameters are the same as in Tables 1 and 2. Growth rate is A) 0 µm^3^s^−1^; B) 25 µm^3^s^−1^; C) 50 µm^3^s^−1^.

Figure 11 shows that, when growth rate increases and the surface to volume ratio decreases simultaneously following 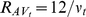
µm^−1^ at the tip, both oscillatory frequency and amplitude in calcium concentration decrease (if 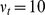
µm^3^, 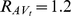 µm^−1^, as shown in Table 2). Experimentally, it has been observed that oscillations in non-growing pollen tubes are with a shorter period [30]. Therefore, this modelling result is qualitatively in agreement with experimental observations. However, quantitatively, the period experimentally observed in a non-growing pollen tube (∼6–7s) is much shorter than that shown in Figure 11. Our model has only considered the effects of growth rate on the volume and the surface (that is occupied by transporters) to volume ratio. The differences between the modelling results and the experimental observations indicate that the growth of a pollen tube may also affect other factors which affect oscillatory period.

Therefore, model analysis predicts that effects of growth rate on ion dynamics is related to its effects on the surface to volume ratio. Experimental analysis on how growth rate is related to the surface to volume ratio will help in elucidating the ion dynamics in pollen tube growth.

### Effects of time delay between ion concentrations and growth rate on ion dynamics

Equation (3) implies that there is an instantaneous response of growth rate to ion concentrations. However, in a cell, the link between ion concentrations and growth rate involves multiple levels of interactions which include gene expression, signalling and metabolism. Therefore, the link between ion concentrations and growth rate may involve time delay. We have therefore examined the effects of time delay between ion concentrations and growth rate on ion dynamics.

**Figure 12:**
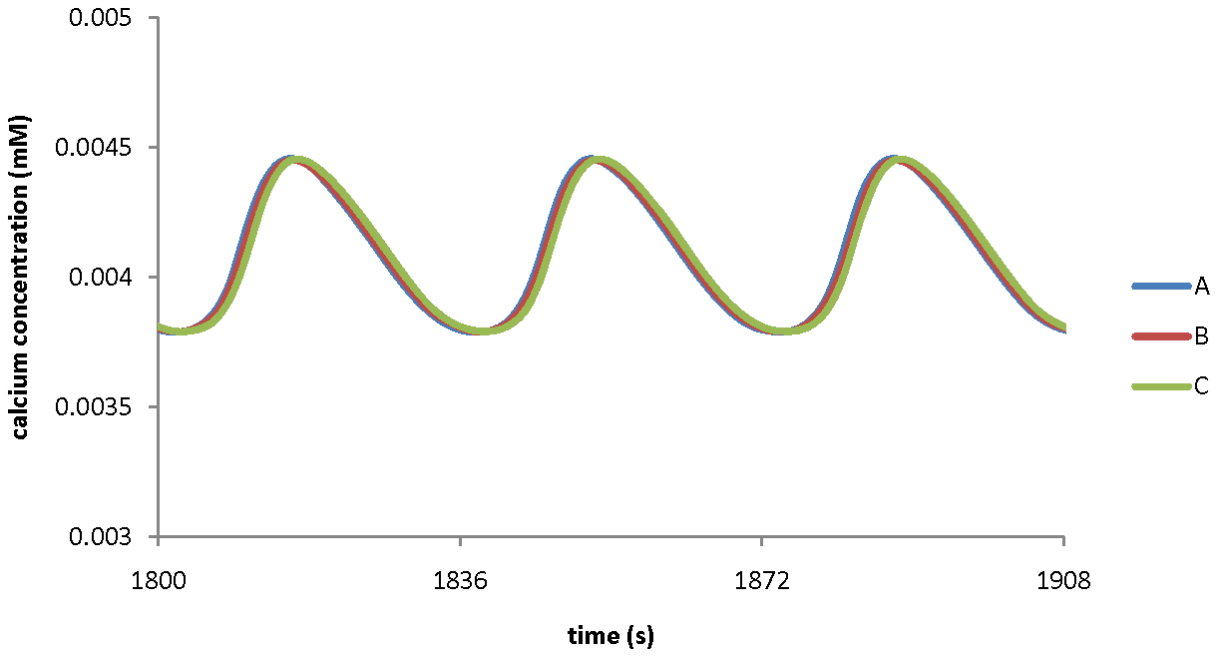
Effects of time delay between ion concentrations and growth rate on the ion dynamics. Time delay: A). 0s; B) 100s; C) 200s. All other parameters are the same as in Figure 8.

In order to examine the effects of time delay, we change equation (3) into
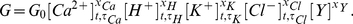
(6)Where τ_Ca_, τ_H_, τ_K_, τ_Cl_ are the delay time for 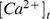, 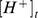, 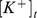, and 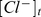 respectively. For example, when 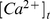 changes, it take 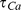 s to realise its effects on growth rate. We have tested that, for 0s≤τ_Ca_≤200s, 0s≤τ_H_≤200s, 0s≤τ_K_≤200s, 0s≤τ_Cl_≤200s, any combination of the delay time has small effects on ion dynamics. Figure 12 shows the results for τ_Ca_ = τ_H_ = τ_K_ = τ_Cl_ = 0s, 100s, 200s, respectively.

## Discussion

By integrating the properties of transporters at both tip and shank of the pollen tube, we have developed a two-compartment model for the tip-shank interactions in this system in the pollen tube. We show that the model is able to establish ion gradients between the tip and shank, with a feature of oscillating dynamics at the tip and a steady state at the shank. Moreover, using the same set of parameters (Tables 1 and 2), we show that our modelling results are also in good agreement with experimental observations a) that polarised ion fluxes are established between the tip and shank; b) that the abundance of H^+^ ATPase transporters at the tip is much lower than that at shank [15]; c) that the period and amplitude of calcium oscillations respond qualitatively to different external ion concentrations (calcium, potassium and pH [26]). Therefore, the features of polarised growth in pollen tubes can be explained by the localised distribution of transporters in the tip and shank compartments.

Model analysis further reveals that ions at both tip and shank form an integrative and self-regulatory dynamical system and they respond to perturbations in a co-coordinated way. Therefore, when any perturbation experiment is designed or when experimental data are explained, this integrative response strategy has to be taken into account. Furthermore, model analysis reveals that oscillatory dynamics at the tip are not important for establishing ion gradients. This is of interest, as a recent paper revealed that apical Ca^2+^ oscillations were not present in pollen tubes growing *in vivo* [31], suggesting that although they have long been considered to be a key feature of tip growth, they are not absolutely required for growth.

Our modelling analysis and the recent experimental observations of Iwano et al. [31] raise further questions about the *in vivo* ion and growth dynamics of pollen tubes. Further model development should take into account the *in vivo* cellular environment. Previous modelling analysis demonstrated that a 5-transporter model [8] behaved differently in small apoplastic volumes than in isolated cells in large volumes of external medium with constant ion concentrations. Additionally, further experimental design should investigate the key factors that cause the differences between *in vitro* and *in vivo* ion and growth dynamics in the pollen tube.

The model suggests that a current travelling along a pollen tube plays a role in self-regulating ion concentrations and in regulating the properties of ion dynamics. These results suggest that in future, experiments designed to understand the underlying mechanism of pollen tube dynamics consideration should be made to uncouple oscillatory dynamics from ion gradients.

Further analysis of the model relating to the coupling between pollen tube growth rate and ion dynamics reveals two possible mechanisms for the growth-induced oscillations: one is due to the transition of tip membrane into shank membrane; the other is due to the effects of growth rate on the kinetic parameters of the transporters. Mechanisms for oscillations in GTPase related growth have been suggested involving the cyclical activation and suppression of small GTPases [32] and proton regulated actin dynamics at the tip [33]. Our modelling analysis shows that growth induced oscillations in all four major ions emerge in an integrative manner with phase differences (see also Data S4 in the Supporting Information for details). Further elucidation of oscillations in factors that effect growth at a systemic level should expand the current modelling framework further to include the dynamics of other functional modules such as GTPases and actin dynamics.

Following previous model developments in plant cells [8]–[10], the development of the model described here is based on two types of relationship between currents and voltages. An Ohmic law is used to describe the current density though pumps and Cl^−^-2H^+^ symporter, while the Goldman-Hodgkin-Katz constant-field current equation is used for all other channels. The current model has established the methodology to integrate the properties of transporters with any current-voltage relationship into compartmental models. For example, stretch-activated Ca^2+^ channels in lily pollen grain and tube tip protoplasts have been experimentally identified [34]. By replacing 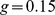 S m^−2^ mM^−1^ for the calcium channel at the tip with 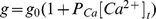 where 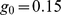 S m^−2^ mM^−1^ and 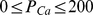
mM^−1^, our numerical analysis shows that all conclusions in this work are valid. In the future, in order to investigate how stretch-activated Ca^2+^ channels affect ion dynamics, the effects of stretch-activated Ca^2+^ channels can be incorporated into the kinetics of Ca^2+^ channels in detail.

From a rigorous point of view, our model is far from being complete for describing pollen tube growth. All parameters are unknown in the model. In this work, the parameters were chosen using the following two criteria (see Materials and Methods): a) calcium concentrations at tip and shank are approximately 0.004 mM and 7.5×10^−5^mM, respectively; and pH at the tip and shank are 6.6 and 7.5, respectively. b) oscillations emerge at the tip with a period of approximately 40s. These parameters are included in Tables 1 and 2, and they are fixed throughout this work with the exception where the effects of varying a parameter value are examined. Our numerical simulation also reveals that, for the parameters used, if ions are not allowed to exchange between the tip and shank, there are no oscillations at the tip. Due to the complexity of this model and the large number of parameters, a comprehensive dynamical analysis of our model presents a challenging future task. However, the agreement of our modelling analysis with the existing experimental data renders our theoretical treatment very promising.

Pollen tube growth is regulated by a wide range of spatiotemporally organised signalling networks and functions including exocytosis and endocytosis, actin cytoskeleton reorganization, cell wall deposition and assembly, phospholipid and inositol polyphosphate signalling, small G-proteins, fertilization, and self-incompatibility [35]. The model developed here in this paper is the first attempt to model a) the dynamics of all four major ions (Ca^2+^, K^+^, H^+^ and Cl^−^) arisen from the interactions between transporters at the tip and shank and b) the interactions between ion dynamics of all four major ions (Ca^2+^, K^+^, H^+^ and Cl^−^) and pollen tube growth. The experiments that examine tobacco pollen tube growth show that hypotonic treatment induces growth rate and apical volume oscillation frequencies change, and they provide strong evidence that hydrodynamic oscillations are closely correlated with or form the basis of the pollen tube oscillator that drives growth rate oscillations and oscillations in ion fluxes and concentrations [21], [36]. Moreover, a model based on physical variables (pressure, surface tension, density and viscosity) and their dependences on calcium concentration and the thickness of cell walls show that a calcium dependent vesicle recycling mechanism is necessary for generating oscillations in growth rate [37]. In addition, model analysis also shows that growth rate oscillations can be generated by the processes involving the cyclical activation and suppression of small GTPases [32]. A finding of our modelling analysis is that oscillations in growth rate of a pollen tube can induce oscillations in ions *via* a) a volume transition from the tip to the shank or b) growth-induced changes in kinetic parameters of ion transporters, therefore our modelling result support the argument that growth rate oscillation driven by hydrodynamic oscillation can be a source of oscillation in ion concentrations and fluxes. In addition, our model analysis shows that a time delay of the interactions between ion concentrations and growth rate is not an important factor affecting the oscillations at the tip. Therefore, when oscillations in growth rate are generated by any mechanism that includes hydrodynamic oscillation, calcium dependent vesicle recycling mechanism, and the cyclical activation and suppression of small GTPases, they can be a source for the oscillations in ion concentrations and fluxes.

As demonstrated here, compartmental models have the advantage of capturing the main properties of biologically defined compartments and furthermore, allow the study of the interactions between compartments (See Data S1 in the Supporting Information for more details). The current two-compartment model presented here has captured the main features of the tip-shank interactions in pollen tube growth and revealed some important aspects of pollen tube growth dynamics. In addition, the current model is expandable and can integrate any number of transporters and any number of compartments. In addition, based on the current model, a spatial model can be developed by introducing the distribution of transporters along the pollen tube, the explicit spatial structure of the pollen tube and the relationship between the distribution and kinetics of the transporters. Currently, the distribution of only a limited number of transporters along the pollen tube is experimentally known, and the effects of the distribution on the kinetics of transporters along the pollen tube are unknown. Therefore further modelling and experimental development should integrate a) explicit spatial settings within each compartment; b) interactions between mechanical properties of membrane and ion dynamics e.g. the relationship between thickness of the membrane and calcium ion concentration [37], [38]; c) the molecular basis of growth as it has been shown that in microorganisms, it is possible to describe the accumulation of biomass in terms of the underlying biological components and processes [39].

The very nature of compartmental models allows the integration of other biologically functioning modules. For example, the establishment of a quantitative relationship between the abundance of a transporter and the ion fluxes it controls may link ion dynamics with the gene expression profile of this transporter. Therefore modelling in this way provides a platform on which to build layers of interacting functional modules. This will be valuable for future studies investigating pollen tube and other tip growth systems. Moreover, the methodology and principles developed here are applicable to the study of ion dynamics and their interactions with other functional modules in any plant cellular system.

## Materials and Methods

### Reversal potentials

The reversal potentials for the four major ions (potassium, proton, calcium, and chloride) are
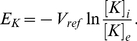
(e1)

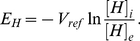
(e2)

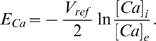
(e3)

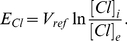
(e4)Where 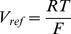 with R is Boltzmann constant, T is temperature, and F is Faraday constant. The subscript “i” indicates intracellular concentrations; the subscript “e” indicates extracellular concentrations.

### Current-voltage relationship

The current density through each transporter for a total of 12 transporters described in Figure 1 is described using two types of current-voltage relationship following previous work [8], [9].

Following previous modelling developments [8]–[10], for transporters t4, t5, s3, s4 and s5 in Figure 1, ohmic relationship (equation (e5)) is used.
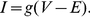
(e5)For transporters t1, t2, t3, t6, s1, s2 and s6 in Figure 1 in the main text, Goldman-Hodgkin-Katz constant-field relationship (equation (e6)) is used.
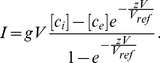
(e6)In equations (e5) and (e6), g is membrane conductance, V is membrane voltage, and E is reversal potential, as described by equations (e1)–(e4), z is the charge of an ion, 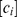 and 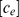 are the intracellular and extracellular ion concentrations, respectively.

### Voltage gating

Voltage gating of all transporters apart from t6 and s6 in Figure 1 in the main text are described using the scheme described in equation (e7) [7], [8].
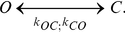
(e7)Where O and C are the completely open and completely closed state, 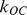 and 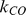 are the rate constants that control the transition between the open (O) and the closed (C) state, and they are functions of membrane voltage. They are described using equation (e8) [8], [9].
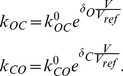
(e8)Where 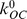 and 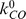 are the rate constants at zero voltage, 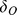 and 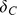 are the voltage-sensitivity coefficients for the open and closed state, respectively. Following previous model developments [8], [9], 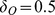 and 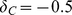.

Voltage gating is described using equation (e9) [7], [8].
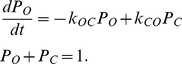
(e9)Where 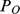 and 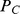 are the probability for the transporter to be at the open and closed state, respectively.

For transporters t6 and s6 (chloride channel) in Figure 1, voltage gating are described using the scheme described in equation (9) [7], [8].
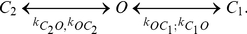
(e10)

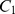
 and 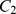 are two different closed states. Voltage gating is described using equation (e11).
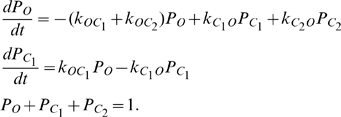
(e11)


### Membrane voltage at tip and shank

It has been shown experimentally that there is a net current intensity (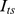) entering the tip and leaving at the tube and grain [14]. Theoretically this net current must be due to the exchange of ions between the tip and the shank, and it is incorporated into the model in the following form,
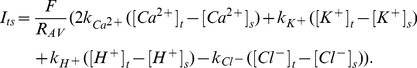
(e12)where F is the Faraday constant, 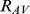 is the surface area to volume ratio at the interface between tip and shank. 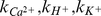 and 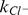 are the rate constants for exchanging Ca^2+^, H^+^, K^+^, and Cl^−^ between tip and shank, respectively. 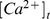, 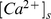, 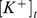, 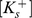
_,_ 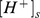, 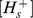 and 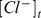, 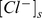 are the respective concentrations of 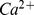, 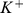, 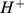 and 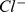 at the tip and shank.

Accordingly, membrane voltage at the tip and shank is calculated by integrating equations (e13) and (e14) respectively.
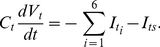
(e13)

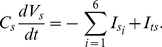
(e14)Where 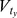 and 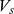 are the membrane voltage at the tip and shank, respectively. 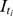 and 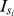 (i = 1,…6) are the currents obtained through the six transporters at the tip and shank, respectively (figure 1). They are calculated following equations (e5) and (e6). 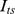 is described in equation (e1). 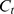 and 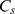 are membrane capacitance at tip and shank respectively. In plant cells, membrane capacitance may be negligible [8], [9]. In this work, for the sake of generality and simplicity, we assume that membrane capacitance is small (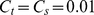 Fm^−2^). We have tested that all conclusions drawn in this work hold for the range of 0–1 Fm^−2^ for membrane capacitance values (other parameters are the same as in Tables 1 and 2).

### Parameters

Parameters were chosen using the following two criteria: a) calcium concentrations at tip and shank are approximately 0.004 mM and 7.5×10^−5^mM, respectively; and pH at the tip and shank are 6.6 and 7.5, respectively. b) oscillations emerge at the tip with a period of approximately 40s. In addition to the emergence of oscillatory dynamics at the tip, our choices of parameters have also taken the following experimental data into account. Using fura 2 dextran and aequorin [11], it is shown that the peak calcium concentration at the tip plasma membrane is between 0.001 and 0.01 mM in the lily pollen tubes. In the ‘steady-state’ shank zone (approximately 20 microns from the tip) the same methods recorded a value between 1.5×10^−4^ and 3×10^−4^ mM in the lily pollen tubes [11]. The concentration of protons within the cytosol has been found to be pH 6.8 at the tip and pH 7.5 within the subapical zone in the lily and tobacco pollen tubes [5], [24]. Our parameters are included in Tables 1 and 2, and they are fixed throughout this work with the exception where the effects of varying a parameter value are examined. We note that the alkaline band (pH = 7.5) is only confined within the subapical zone in the lily and tobacco pollen tubes [5], [24]. The distal region of the shank is also with a lower pH [5]. Our parameters are aimed at generating the alkaline band in the shank. The other regions of the shank are described by a body compartment, as described in DATA S1 in the Supporting Information.

### Buffering of H^+^ and Ca^2+^

As shown in Figure 1 in the main text, buffering of H^+^ and Ca^2+^ are introduced at both the tip and shank using the following generic reactions.
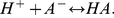
(e15)

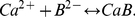
(e16)The equilibrium of reaction (e15) is described by 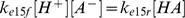. The equilibrium of reaction (e16) is described by 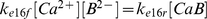. At both tip and shank, the equilibrium constant for each of the two reactions is set to be the same, as expected for a chemical reaction. At both tip and shank, 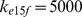
mM^−1^s^−1^, 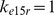
s^−1^, the equilibrium constant 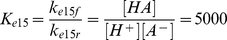
mM^−1^. Similarly, 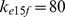
mM^−1^s^−1^, 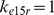
s^−1^, the equilibrium constant 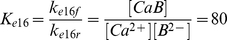
mM^−1^ at both the tip and shank. The input and output of 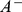, 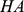, 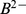 and 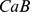 at the tip and shank include the overall contribution of all the following processes: a) their production and consumption due to other biological processes; b) their exchange between the tip and shank; c) their exchange with extracellular space; d) their exchange between the shank and the body compartment. In addition, the cell wall may absorb a large portion of calcium [11]. In our model, it is assumed that such an effect of the cell wall on calcium concentration is already represented in the buffering reactions and medium compositions (i.e. extracellular ion concentrations). The input and output rates of 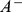, 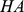, 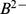 and 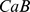 at both the tip and shank are assumed to follow zero-order and first-order kinetics. At the tip: 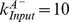
mMs^−1^, 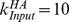
s^−1^, 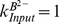
mMs^−1^, 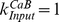
s^−1^, 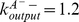
s^−1^, 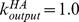
s^−1^, 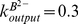
s^−1^, 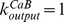
s^−1^, respectively. At the shank: 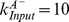
mMs^−1^, 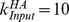
s^−1^, 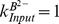
mMs^−1^, 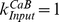
s^−1^, 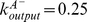
s^−1^, 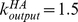
s^−1^, 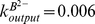
s^−1^, 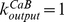
s^−1^, respectively. The buffering reactions (e15) and (e16) are incorporated into ion dynamics (equations (1) and (2)) in the form of equations (e17) and (e18) with 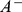, 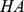, 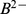 and 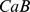 being calculated based on the mass balance as described above.
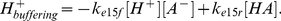
(e17)

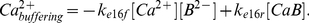
(e18)Apart from the transport of H^+^ and Ca^2+^ through membrane at tip and shank and the exchange of H^+^ and Ca^2+^ between tip and shank, all other factors affecting H^+^ and Ca^2+^ concentrations at both tip and shank are incorporated into the buffering reactions. Therefore, the buffering reactions (e15) and (e16) may include a number of reactions, which can be different at the tip and shank. When the buffering compounds (A^−^, HA, B^2−^, CaB) are experimentally known, these buffering reactions can be explicitly expanded. Although this model only includes the buffering of H^+^ and Ca^2+^, the methodology for introducing buffering is generic and the buffering of K^+^ and Cl^−^ can be easily introduced for the compartmental model if it is required.

### Numerical Analysis

The model is programmed using Berkeley Madonna (www.berkeleymadonna.com). The Rosenbrock (Stiff) method is used to integrate the differential equations with a tolerance of 1.0E-7. The codes can be made available on request (Junli.liu@durham.ac.uk). The numerical results in published models [7], [8] were exactly reproduced using the same settings.

## Supporting Information

Data S1 Principle for developing compartmental models.
(0.21 MB DOC)

Data S2 Integrative ion dynamics for responding to intracellular perturbations.
(3.85 MB DOC)

Data S3 Abstraction of the two-compartment model into an electrical dipole circuit.
(0.06 MB DOC)

Data S4 Phase Shifts
(0.94 MB DOC)
