# Supplementary material for: A Compartmental Model Analysis of Integrative and Self-Regulatory Ion Dynamics in Pollen Tube Growth
Source: PLoS One. 2010 Oct 6;5(10):e13157. doi: 10.1371/journal.pone.0013157 (PMC2950844; doi:10.1371/journal.pone.0013157)
Supplement: Data S1 — Principle for developing compartmental models. (0.21 MB DOC) [file pone.0013157.s001.doc]

**Principle for developing compartmental models in pollen tube growth**

The basic principle for developing compartmental models in biology is to capture the main properties of biologically defined compartments and to study the interactions between them. The advantage for developing compartmental models is that compartmental models are able to establish close relationship with experimental designs: model development is based on the knowledge and data of biologically defined compartments and modelling results can be tested using experimental designs based on compartments. For pollen tube, three biologically defined compartments can be defined, and they are tip, shank and body.

For the three compartments, the mass balance of four major ions is described as follows.

At tip

$\begin{matrix} \frac{d[Ca^{2+}]_{t}}{dt}=-\frac{R_{AV_{t}}I_{t3}}{2F}-k_{Ca^{2+}}([Ca^{2+}]_{t}-[Ca^{2+}]_{s})-\mu_{t}[Ca^{2+}]_{t}+Ca_{buffering}^{2+} \\ \frac{d[H^{+}]_{t}}{dt}=-\frac{R_{AV_{t}}(I_{t4}+2I_{t5})}{F}-k_{H^{+}}(H^{+}]_{t}-[H^{+}]{}_{s})-\mu_{t}[H^{+}]_{t}+H_{buffering}^{+} \\ \frac{d[K^{+}]_{t}}{dt}=-\frac{R_{AV_{t}}(I_{t1}+I_{t2})}{F}-k_{K^{+}}([K^{+}]_{t}-[K^{+}]_{s})-\mu_{t}[K^{+}]_{t} \\ \frac{d[Cl^{-}]_{t}}{dt}=-\frac{R_{AV_{t}}(-I_{t6}+I_{t5})}{F}-k_{Cl^{-}}([Cl^{-}]_{t}-[Cl^{-}]_{s})-\mu_{t}[Cl^{-}]_{t}. \end{matrix}$ (1)

At shank,

$\begin{matrix} \frac{d[Ca^{2+}]_{s}}{dt}=-\frac{R_{AV_{s}}(I_{s2}+I_{s3})}{2F}+k_{Ca^{2+}}\mu_{ts}([Ca^{2+}]_{t}-[Ca^{2+}]_{s})-\mu_{s}[Ca^{2+}]_{s}+Ca_{buffering}^{2+}-k_{Ca^{2+}}([Ca^{2+}]_{s}-[Ca^{2+}]_{b}) \\ \frac{d[H^{+}]_{s}}{dt}=-\frac{R_{AV_{s}}(I_{s4}+2I_{s5})}{F}+k_{H^{+}}\mu_{ts}([H^{+}]_{t}-[H^{+}]_{s})-\mu_{s}[H^{+}]_{s}+H_{buffering}^{+}-k_{H^{+}}([H^{+}]_{s}-[H^{+}]_{b}) \\ \frac{d[K^{+}]_{s}}{dt}=-\frac{R{}_{AV_{s}}(I_{s1})}{F}+k_{K^{+}}\mu_{ts}([K^{+}]_{t}-[K^{+}]_{s})-\mu_{s}[K^{+}]_{s}-k_{K^{+}}([K^{+}]_{s}-[K^{+}]_{b}) \\ \frac{d[Cl^{-}]{}_{s}}{dt}=-\frac{R{}_{AV_{s}}(-I_{s6}+I_{s5})}{F}+k_{Cl^{-}}\mu_{ts}([Cl^{-}]_{t}-[Cl^{-}]_{s})-\mu_{s}[Cl^{-}]_{s}-k_{Cl^{-}}([Cl^{-}]_{s}-[Cl^{-}]_{b}). \end{matrix}$ (2)

At body,

$\begin{matrix} \frac{d[Ca^{2+}]_{b}}{dt}=k_{Ca^{2+}}\mu_{sb}([Ca^{2+}]_{s}-[Ca^{2+}]_{b})-\mu_{b}[Ca^{2+}]_{b}+Ca_{ib}^{+2}-Ca_{eb}^{+2} \\ \frac{d[H^{+}]_{b}}{dt}=k_{H^{+}}\mu_{sb}([H^{+}]_{s}-[H^{+}]_{b})-\mu_{b}[H^{+}]_{b}+H_{ib}^{+}-H_{eb}^{+} \\ \frac{d[K^{+}]_{b}}{dt}=k_{K^{+}}\mu_{sb}([K^{+}]_{s}-[K^{+}]_{b})-\mu_{b}[K^{+}]_{b}+K_{ib}^{+}-K_{eb}^{+} \\ \frac{d[Cl^{-}]{}_{b}}{dt}=k_{Cl^{-}}\mu_{sb}([Cl^{-}]_{s}-[Cl^{-}]_{b})-\mu_{b}[Cl^{-}]_{b}+Cl_{ib}^{-}-Cl_{eb}^{-}. \end{matrix}$(3)

In the right hand of equations (1) and (2), the first term describes the transport of ions through membrane; the second term describes the exchange of ions between tip and shank; the third term describes the effects of volume changes on ion concentration. For Ca^2+^ and H^+^, the fourth term ($Ca_{buffering}^{2+}$or $H_{buffering}^{+}$) describes the buffering of Ca^2+^ and H^+^ , respectively. $R{}_{AV_{t}}$ and $R_{AV_{s}}$are the surface to volume ratio for describing the density of transporters at the membrane surface of tip and shank compartments, and they are set to be equal throughout this work. $\mu_{ts}$ is the volume ratio between tip and shank. In equation (2), the last term describes the exchange of ions between shank and body. In equation (3), the first term describes the exchange of ions between shank and body, the second term describes the effects of volume changes on ion concentration in body compartment, and the last two terms describe the influx and efflux of ions in the body compartment. The biological knowledge about the last two terms is scarce. $\mu_{sb}$is the volume ratio between body and shank.

When a pollen tube grows, new membranes emerge at the tip first, then convert into shank volumes, then to body volumes. Therefore, the volume changes in each compartment are generally described as follows.

$\begin{matrix} \frac{d\nu_{t}}{dt}=f_{t} \\ \frac{d\nu_{s}}{dt}=f_{s} \\ \frac{d\nu_{b}}{dt}=f_{\begin{matrix} b \\ \end{matrix}} \\ f_{t}+f_{s}+f{}_{b}=G. \end{matrix}$ (4)

Where $\nu_{t}$, $\nu_{s}$ and $\nu_{b}$are the volume for tip , shank and body compartment, respectively. $G$is growth rate.

The net current from tip to shank and from shank to body is due to the exchange of ions between tip and shank, and between shank and body, respectively. The current is described as follows,

$\begin{matrix} I_{ts}=\frac{F}{R_{AV}}(2k_{Ca^{2+}}([Ca^{2+}]_{t}-[Ca^{2+}]_{s})+k_{K^{+}}([K^{+}]_{t}-[K^{+}]_{s})+k_{H^{+}}([H^{+}]_{t}-[H^{+}]_{s})-k_{Cl^{-}}([Cl^{-}]_{t}-[Cl^{-}]_{s})) \\ I_{sb}=\frac{F}{R_{AV}}(2k_{Ca^{2+}}([Ca^{2+}]_{s}-[Ca^{2+}]_{b})+k_{K^{+}}([K^{+}]_{s}-[K^{+}]_{b})+k_{H^{+}}([H^{+}]_{s}-[H^{+}]_{b})-k_{Cl^{-}}([Cl^{-}]_{s}-[Cl^{-}]_{b})). \end{matrix}$ (5)

where F is the Faraday constant, $R_{AV}$ is the surface area to volume ratio at the interface between tip and shank. $k_{Ca^{2+}},k_{H^{+}},k_{K^{+}}$ and $k_{Cl^{-}}$are the rate constants for exchanging Ca^2+^ , H^+^, K^+^, and Cl^-^ between tip and shank, respectively. $[Ca^{2+}]_{t}$, $[Ca^{2+}]_{s}$, $[Ca^{2+}]_{b}$, $[K^{+}]_{t}$, $[K_{s}^{+}]$,$[K_{b}^{+}]$,$[H^{+}]_{s}$, $[H_{s}^{+}]$,$[H_{b}^{+}]$and $[Cl^{-}]_{t}$, $[Cl^{-}]_{s}$,$[Cl^{-}]_{b}$ are the respective concentration of $Ca^{2+}$, $K^{+}$,$H^{+}$and$Cl^{-}$at tip, shank and body.

Accordingly, membrane voltage at tip and shank is calculated by integrating equations (21) and (22) respectively.

$C_{t}\frac{dV_{t}}{dt}=-(\sum_{i=1}^{6} I_{ti}+I_{ts}).$ (6)

$C_{s}\frac{dV_{s}}{dt}=-(\sum_{i=1}^{6} I_{si}-I_{ts}+I_{sb}).$ (7)

Where $V_{t_{y}}$ and $V_{s}$ are the membrane voltage at tip and shank, respectively. $I_{ti}$ and $I_{si}$ (i=1,...6) is the current via the six transporters at the tip and shank, respectively. $I_{ts}$and$I_{sb}$are described in equation (1). $C_{t}$ and $C_{s}$are membrane capacitance at tip and shank respectively. For simplicity, we assume that $C_{t}=C_{s}$.

When a pollen tube grows, its growth rate is related to many unknown factors. As far as the ions involved are concerned, it is reasonable to assume that the growth rate is related to all major ions, but the quantitative relationship between the ion concentrations and the growth rate are largely unknown. Therefore, in this work, we propose to use a power-law formalism to describe the relationship between the ion concentrations and the growth rate. the power-law formalism or S-system theory was developed by Savageau et. al [1,2] and it is a useful methodology to describe complex biological interactions, in particular when detailed kinetic knowledge of the underlying biological processes is unknown [1,2]. Different types of kinetic equations can be re-casted into power-law formalism [2]. Following power-law formalism, growth rate can be generally expressed as

$G=G_{0}[Ca^{2+}]_{t}^{x_{Ca}}[H^{+}]_{t}^{x_{H}}[K^{+}]_{t}^{x_{K}}[Cl^{-}]_{t}^{x_{Cl}}[Y]^{x_{Y}}.$ (8)

In equation (23), $G$is growth rate, and $G_{0}$is a constant. $x_{Ca},x_{H},x_{K},x_{Cl}$and $x_{Y}$ are the powers for four ions at tip and an unknown factor Y, and these powers can take any negative and positive values or zero. All concentrations are in the unit of mM. Using equation (8), growth rate is coupled with ion dynamics. Since the quantitative relationship between growth rate and ion concentrations is largely unknown, for simplicity, we take $x_{Ca}=1$, $x_{H}=1$, $x_{K}=1$, $x_{Cl}=1$ and $x_{Y}=1$. $G_{0}=20-2000$μm^3^/(s mM^5^) leads to a growth rate to be ~0.01-1 μm^3^/s for [Y]= 1 mM and all other parameters in Tables 1 and 2 in the main text.

It is experimentally evident that both tip and shank compartments of a pollen tube approximately keep constant volumes when a pollen tube grows, and body compartment increases its volume. Therefore, we set $f_{t}=0$, $f_{s}=0$and $f_{b}=G$. Since the influx and efflux data at body compartment are experimentally scarce, we assume both influx and efflux of all four ions are zero. We have numerically examined the 3-compartment model using $G_{0}=20-2000$μm^3^/(s mM^5^) , which corresponds to a growth rate of ~0.01-1 μm^3^/s, and found that the respective concentration of the four ions in shank and body compartment always establishes a quasi-equilibrium state (for the quasi-equilibrium state, the respective concentration difference of all four ions is always less than 1%).

Based on equations (1-8), we know that, when the respective concentration of ions in shank and body compartment is in equilibrium, tip and shank compartments can be decoupled from body compartment in the model development. Therefore a tip-shank two-compartment model can be developed. For the two-compartment model, equation (1) remains and in equation (2) the last term for all four ions becomes zero.

The mass balance of all ions in 3-compartment model is maintained and it is described as follows. For example, for $Ca^{2+}$,

$\frac{dm_{Ca^{2+}}}{dt}=-\frac{\nu_{t}R_{AV_{t}}I_{t3}}{2F}+\nu_{t}Ca_{buffering}^{2+}-\frac{\nu_{s}R_{AV_{s}}(I_{s2}+I_{s3})}{2F}+\nu_{s}Ca_{buffering}^{2+}+\nu_{b}(Ca_{ib}^{+2}-Ca_{eb}^{+2}).$ (9)

where $m_{Ca^{2+}}$ is in the unit of mmol.

Equation (9) indicates that the mass change in a pollen tube is due to $Ca^{2+}$influxes and effluxes at all three compartments. The mass balance for other ions can be derived in a similar manner.

For the 2-compartment model, the mass balance of all ions is maintained and it is described as follows. For example, for $Ca^{2+}$,

$\frac{dm_{Ca^{2+}}}{dt}=-\frac{\nu_{t}R_{AV_{t}}I_{t3}}{2F}+\nu_{t}Ca_{buffering}^{2+}-\frac{\nu_{s}R_{AV_{s}}(I_{s2}+I_{s3})}{2F}+\nu_{s}Ca_{buffering}^{2+}+G[Ca^{2+}]_{s}.$ (10)

Equation (10) indicates that the mass change in a pollen tube is due to $Ca^{2+}$influxes and effluxes at both tip and shank compartments and consumption of $Ca^{2+}$for growth in body compartment (note that $Ca^{2+}$concentration is assumed to be the same at shank and body). The mass balance for other ions can be derived in a similar manner.

The only difference between a 3-compartment model and a 2-compartment model is that, in the 2-compartment model, the respective ion concentration in shank and body compartment is assumed to be the same (ie. the respective concentration of ions is in equilibrium between shank and body compartment) when a pollen tube grows. Therefore, the net current from shank to body ($I_{sb}$) is zero. As discussed above, this assumption can be held when the influx and efflux of all ions at body compartment set to be zero. We have numerically tested the effects of the body compartment, finding that inclusion of the body compartment does not affect our conclusions for a wide range of influx and efflux of ions at body compartment. By considering that little is known about biological information in relation to body compartment, this work studies the ion dynamics of pollen tube by comprehensively developing the tip-shank 2-compartment model.

1. Savageau MA (1976) Biochemical systems analysis: a study of function and design in molecular biology, Addison-Wesley, Reading, Mass.
2. Voit EO (1991) (ed) Canonical non-linear modeling: S-system approach to understanding complexity. New York. Van Nostrand Reinhold.
