# Supplementary material for: A Compartmental Model Analysis of Integrative and Self-Regulatory Ion Dynamics in Pollen Tube Growth
Source: PLoS One. 2010 Oct 6;5(10):e13157. doi: 10.1371/journal.pone.0013157 (PMC2950844; doi:10.1371/journal.pone.0013157)
Supplement: Data S2 — Integrative ion dynamics for responding to intracellular perturbations. (3.85 MB DOC) [file pone.0013157.s002.doc]

**Integrative ion dynamics for responding to intracellular perturbations**

Figure 1 shows an example, for which there is an extra source for producing chloride with a rate of 0.3 mM/s between 1000s and 2500s. After 2500s, this extra source is removed. This kind of perturbation is biologically equivalent to the perturbations introduced by intracellular biological processes.


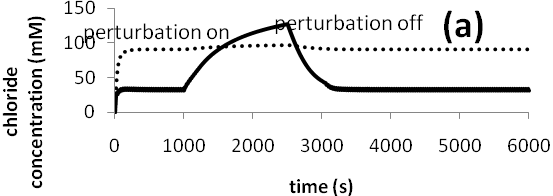


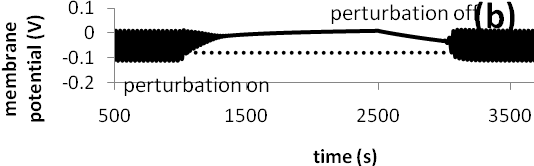


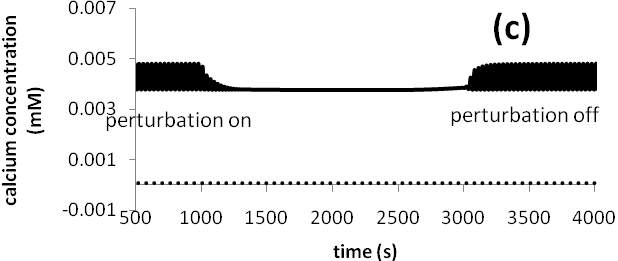

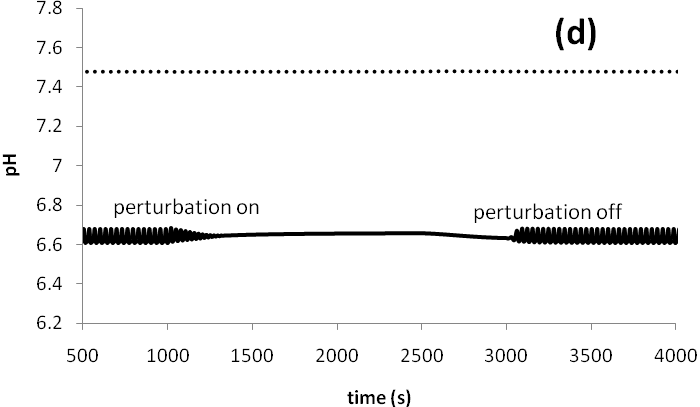


Figure 1. Integrative ion dynamics for responding to intracellular chloride perturbations. At 1000s, the perturbation is superimposed with a rate of 0.3 mM/s. At 2500s, this perturbation is removed.

Figure 1 shows that, when an extra source for producing chloride with a rate of 0.3 mM/s between 1000s and 2500s is added, membrane potentials at both tip and shank (figure 1b) change. Once membrane voltages change, they act as “global regulators” regulating the kinetic properties of all channels and pumps at both tip and shank. the probabilities for all transporters to be at open or closed state change accordingly. Consequently, the currents relating to all transporters change. These changes in the currents lead to the changes in four ion concentrations. Figures 1c and 1d show the response of calcium concentration and PH. In turn, changes in the ion concentrations again contribute to changes in currents and voltages by a) changing the currents through membrane at both tip and shank; and b) by changing ion gradients that lead to the changes in the current travelling along pollen tube. Therefore, following the perturbation to internal chloride production rate, the tip and shank respond as an integrative dynamical system, resulting in a coordinated response.

Therefore, although internal and external perturbations may be realised using different biological means, the response of pollen tube to internal perturbations (figure 1) follows the same underlying mechanism as its response to external perturbations.

Effects of different initial concentrations of all ions on the ion dynamics are also examined. It reveals that, for randomly generated initial concentrations between 0.1 mM and 50mM for calcium, potassium and chloride and for randomly generated initial PH values between 2 and 9, all four ions always settle onto the same dynamical state after transient periods die out. Therefore, the ion dynamics at tip and shank are independent of the initial conditions. Figure 2 shows an example.


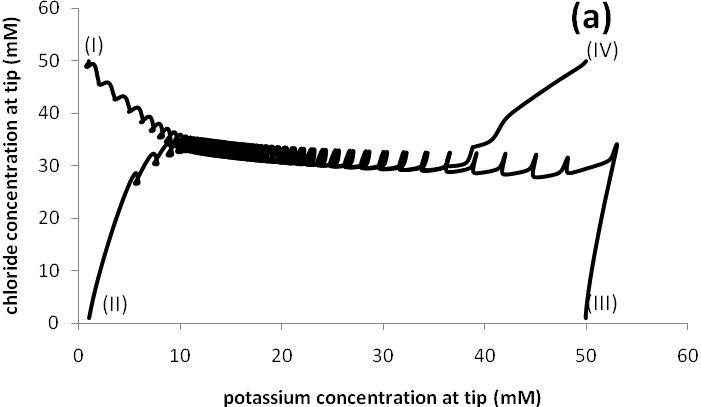


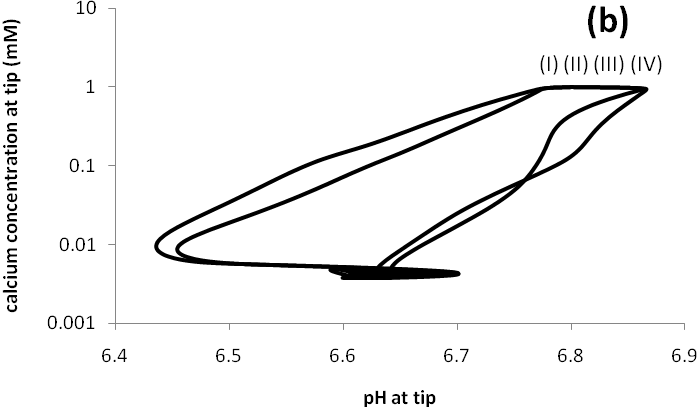


Figure 2. An example shows that the ion dynamics at tip are insensitive to the initial conditions. Initial conditions: (I) $[K^{+}]_{t}=1$mM, $[Cl^{-}]_{t}=50$ mM, $[Ca^{2+}]_{t}=1$ mM, pH_t_$=6.8$. (II) $[K^{+}]_{t}=1$ mM, $[Cl^{-}]_{t}=1$ mM, $[Ca^{2+}]_{t}=1$ mM, pH_t_ $=6.8$. (III) $[K^{+}]_{t}=50$ mM, $[Cl^{-}]_{t}=1$ mM, $[Ca^{2+}]_{t}=1$ mM, pH_t_ $=6.8$. (IV) $[K^{+}]_{t}=50$ mM, $[Cl^{-}]_{t}=50$ mM, $[Ca^{2+}]_{t}=1$ mM, pH_t_$=6.8$. Initial conditions for all ions at shank do not change: $[K^{+}]_{s}=1$ mM, $[Cl^{-}]_{t}=1$ mM, $[Ca^{2+}]_{t}=1$ mM, $PH_{t}=7.5$. (a) evolution on $[K^{+}]_{t}\sim[Cl^{-}]_{t}$ phase plane; (b) the corresponding response on pH_t_ $\sim[Ca^{2+}]_{t}$ phase plane.
