# Supplementary material for: A Compartmental Model Analysis of Integrative and Self-Regulatory Ion Dynamics in Pollen Tube Growth
Source: PLoS One. 2010 Oct 6;5(10):e13157. doi: 10.1371/journal.pone.0013157 (PMC2950844; doi:10.1371/journal.pone.0013157)
Supplement: Data S3 — Abstraction of the two-compartment model into an electrical dipole circuit. (0.06 MB DOC) [file pone.0013157.s003.doc]

**Abstraction of the two-compartment model into an electrical dipole circuit**

Based on experimental observations, a circuit equivalent to an electrical dipole was proposed ([1] and references therein). According to the two-compartment model (figure 1 in the main text), a circuit can be abstracted to describe the current flow for the interaction between tip and shank.


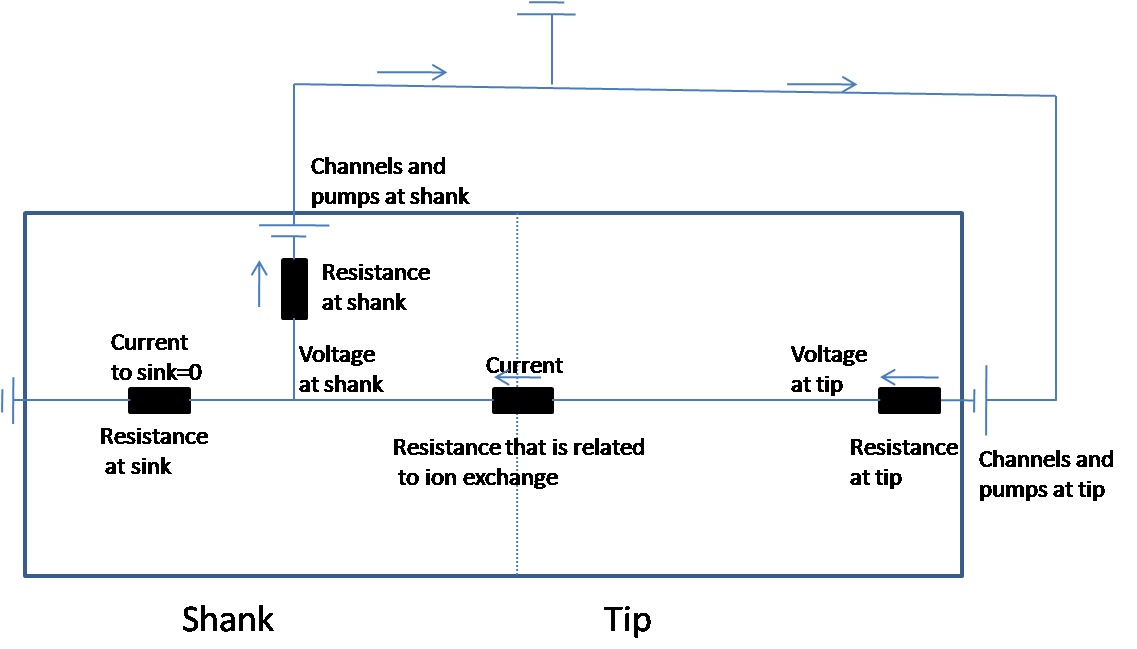


Figure 1. An equivalent electrical dipole circuit of the tip-shank interaction in pollen tube based on the two-compartment model.

In figure 1, the channels and pumps at tip and shank are described by two batteries, implying that the transport of the four ions at tip and shank play a role of a battery. The current between tip and shank is linked with ion gradients. Therefore, the resistance between tip and shank compartment are related to the exchange rates of all four ions.

1. Michard E, Alves F, Feijó JA (2009) The role of ion fluxes in polarized cell growth and morphogenesis: the pollen tube as an experimental paradigm. *Int J Dev Biol* , 53: 1609-1622.
