# Supplementary material for: A Compartmental Model Analysis of Integrative and Self-Regulatory Ion Dynamics in Pollen Tube Growth
Source: PLoS One. 2010 Oct 6;5(10):e13157. doi: 10.1371/journal.pone.0013157 (PMC2950844; doi:10.1371/journal.pone.0013157)
Supplement: Data S4 — Phase Shifts (0.94 MB DOC) [file pone.0013157.s004.doc]

**Phase shifts**

In general, phase shifts in the variables of the model exist for both the intrinsic oscillations generated by ion dynamics and growth-induced oscillations. However, the phase shifts generated using the model and parameters in Tables 1 and 2 in the main text are generally not in good agreement with experimental observations [1,2]. Phase shifts can be adjusted by adjusting parameter values (see Table 1). Figure 1 shows an example for phase shifts in growth-induced oscillations ($k_{OC_{1}}^{0}=0.01$ s^-1^for the chloride channel at tip and all other parameters are the same as in Tables 1 and 2 in the main text).


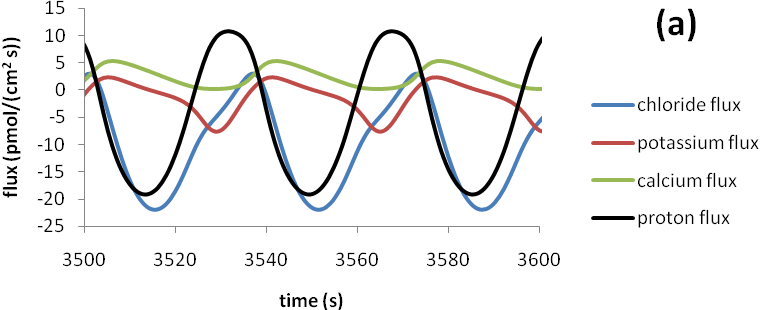


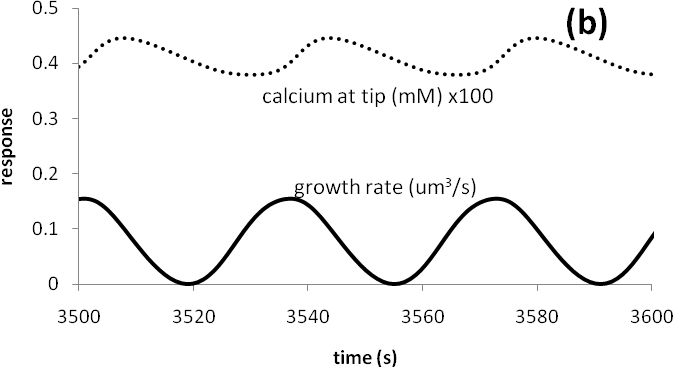


Figure 1. An example for phase shifts in growth-induced oscillations (see main text for details). $k_{OC_{1}}^{0}=0.01$ s^-1^ for the chloride channel at the tip and all other parameters are the same as in Tables 1 and 2 in the main text.

We summarise the phase shifts for three different computational conditions in Table 1: a) Oscillations are generated by ion dynamics (all parameters are the same as in Tables 1 and 2 in the main text) and growth rate is linked with all four major ions (Ca^2+^, K^+^, H^+^ and Cl^-^) using equation (3) in the main text; b) as in a), but we assume growth rate is only linked with K^+^ (ie., $G=G_{0}[K^{+}]^{x_{K}}$ with G_0_=0.02 μm^3^/(s mM), x_K_=1. For these parameters, growth rate is ~0.2 μm^3^s^-1^ ); c) growth-induced oscillations as shown in Figure 1 in this file.

Table 1. Phase shifts for three different computational conditions and experimental observations

| Phase shifts with reference to maximum growth rate | Condition a) | Condition b) | Condition c) | Experimental observations as summarised by Chebli and Geitmann [2] |
| --- | --- | --- | --- | --- |
| Maximum growth rate | 0^o^ | 0^o^ | 0^o^ | 0^o^ |
| Minimum growth rate | 180^o^ | 180^o^ | 180^o^ | 180^o^ |
| Ca^2+^ influx at the tip | 350^o^ | 254^o^ | 50^o^ | 123^o^ [3] or 149^o^ [4] |
| K^+^ influx at the tip | 340^o^ | 348^o^ | 4^o^ | 100^o^ [3] |
| H^+^ influx at the tip | 185^o^ | 90^o^ | 307^o^ | 67.5^o^ [5] or 103^o^ [3] |
| Cl^-^ efflux at the tip | 86^o^ | 354^o^ | 140^o^ |  |
| Maximum [Ca^2+^] at the tip | 0^o^ | 90^o^ | 58^o^ | 38^o^ [6] |
| Maximum [K^+^] at the tip | 90^o^ | 0^o^ | 250^o^ |  |
| Maximum [H^+^] at the tip | 210^o^ | 116^o^ | 130^o^ |  |
| Maximum [Cl^-^] at the tip | 25^o^ | 60^o^ | 260^o^ |  |

As shown in Table 1, for the three conditions, phase shifts change dramatically. Our analysis reveals that the following two factors are important for phase shifts: a) the quantitative relationship between ion concentrations and growth rate; b) the source of oscillations. Growth-induced oscillations may have different phase shifts from those oscillations generated by intrinsic ion dynamics. The equation (3) in the main text describes a general relationship between the concentrations of four major ions (Ca^2+^, K^+^, H^+^ and Cl^-^) and growth rate. A model based on physical variables (pressure, surface tension, density and viscosity) and their dependences on calcium concentration and the thickness of cell walls show that a calcium dependent vesicle recycling mechanism is necessary for generating oscillations in growth rate [7]. Based on the our analysis in Table 1, in order to understand the phase shifts in a growing pollen tube, a quantitative relation between the concentrations of all four major ions(Ca^2+^, K^+^, H^+^ and Cl^-^) and growth rate must be quantitatively established in detail. Kroeger et al. has shown [7] how a relationship between calcium concentration and growth rate can be established based on mechanical principles. This methodology should be further developed to include other ions. As we discussed in the main text, the molecular basis of pollen tube growth should be established as well. As shown in Table 1, the phase shifts computed based on our equation (3) in the main text in our model are largely different from experimental observations. We note that, in general, the oscillations in a growing pollen tube are with irregular amplitudes and frequency [8].

This work has not made efforts to address the differences between experimental and computational phase shifts. Instead, we propose that phase shifts reflect some more subtle aspects of pollen tube growth that require a more comprehensive model development based further on the following observations.

1. Since the changes in kinetic parameters may change phase shifts, growth rate, as a kinetic parameter, has to be quantitatively linked with ion concentrations based on experimental measurements.
2. Since the transition from tip membrane to shank membrane may play a role in the ion dynamics, a quantitative transition rule that reflects the reality of pollen tube growth must be established.
3. Effects of spatial settings in each compartment should be included to examine how inhomogeneous tip and shank compartments affect ion dynamics.
4. Since mechanical properties of cell wall may play a role in the oscillations of pollen tube growth, they should be incorporated into model development in order to quantitatively address phase shifts. A recent model [7] has showed that coupling the thickness of cell wall with calcium concentration only in a tip-growth model may generate oscillations. Interestingly, the phase shifts generated using that model [7] do not agree with experimental observations either.
5. It is inevitable that experimental data are noisy. How do stochastic factors affect phase shifts in developed models should be examined [9,10]before modelled phase shifts are compared with experimental data.
6. Holdaway-Clarke TL, Hepler PK (2003) Control of pollen tube growth: role of ion gradients and fluxes. *New Phytol* 159: 539–563.
7. Chebli Y, Geitmann A (2007) Mechanical principles governing pollen tube growth. *Funct Plant Sci Biotechnol* 1: 232-245.
8. Messerli MA, Danuser G, Robinson KR (1999) Pulsatile influxes of H^+^, K^+^ and Ca^2+^ lag growth pulses of Lilium longiflorum pollen tubes. *J. Cell. Sci.* 112: 1497-1509.
9. Holdaway-Clarke TL, Feijó JA, Hackett GR, Kunkel JG, Hepler PK (1997) Pollen tube growth and the intracellular cytostolic calcium gradient oscillate in phase while extracellular calcium influx is delayed. *Plant Cell* 9: 1999–2010.
10. Messerli MA, Robinson KR (1998) Cytoplasmic acidification and current influx follow growth pulses of Lilium longiflorum pollen tubes. *Plant Journal* 16: 87–91.
11. Messerli MA, Creton R, Jaffe LF, Robinson KR (2000) Periodic increases in elongation rate precede increases in cytosolic Ca2+ during pollen tube growth. *Developmental Biololgy* 222: 84–98.
12. Kroeger JH, Geitmann A, Grant M (2008) Model for calcium dependent oscillatory growth in pollen tubes. *Journal of Theoretical Biology* 253: 363– 374.
13. Michard E, Alves F, Feijó JA (2009) The role of ion fluxes in polarized cell growth and morphogenesis: the pollen tube as an experimental paradigm. *Int J Dev Biol* , 53: 1609-1622.
14. Liu JL, Crawford JW, Viola R, Goodman, B (1997) Prospects for advancing the understanding of complex biochemical systems. *Plant Molecular Biology* 33: 573-581.
15. Turner TE, Schnell S, Burrage K (2004) Stochastic approaches for modelling in vivo reactions. *Comput. Biol. Chem.* 28: 165–178.
